# Functional divergence of protein kinase A regulatory subunit Iβ variants: the importance of N3A motifs in PKA regulation

**DOI:** 10.1111/febs.70358

**Published:** 2025-12-13

**Authors:** Maximilian Wallbott, Jui‐Hung Weng, Valeria Pane, Yuliang Ma, Jian Wu, Susan S. Taylor, Friedrich W. Herberg

**Affiliations:** ^1^ Department of Biochemistry, Institute for Biology University of Kassel Germany; ^2^ Department of Pharmacology University of California CA USA; ^3^ Department of Biochemistry and Molecular Biophysics University of California CA USA

**Keywords:** bioluminescence resonance energy transfer (BRET), cAMP signaling, molecular dynamics, protein kinase, protein kinase A (PKA), regulatory subunit Iβ (RIβ), Surface plasmon resonance spectroscopy

## Abstract

Protein kinase A (PKA) regulatory subunit Iβ (RIβ) plays a crucial role in modulating PKA activity through its interaction with the catalytic (C) subunit. Recent studies have identified two variants of RIβ that have not been distinguished until now. The variants differ in a single residue at Position 268 (alanine vs arginine), located within one of two structural motifs known as N3A motifs. Our study reveals distinct biochemical functions of the variants, highlighting the role of the second N3A motif at the N terminus of the cyclic nucleotide binding domain B (CNB‐B). We demonstrate an enhanced binding affinity of the A268 variant for cAMP and an altered interaction with the C‐subunit. Substitution in the N3A^B^ motif also affects the cooperativity between the cAMP binding sites as well as kinase activity in the absence of cAMP. In HEK293 cells, we demonstrated a reduced cAMP‐induced dissociation of RIβ R268 PKA holoenzymes in a time‐dependent manner. Gaussian molecular dynamics simulations revealed that the CNB‐A domain is more flexible in A268 while the opposite is true for the CNB‐B domain, which is more dynamic in the R268 protein. This study underscores the importance of distinguishing between the two RIβ variants, as they exhibit distinct biochemical properties that alter PKA regulation. Comparison of cellular localization showed that both variants form small droplets in the cytoplasm that colocalize with the C‐subunit in the presence of cAMP. These findings suggest that both RIβ variants undergo liquid–liquid phase separation, similar to RIα.

## Graphical abstract

The regulatory subunit RIβ of protein kinase A occurs in two variants that differ at residue 268 (A268 versus R268). This seemingly small substitution significantly changes cAMP sensitivity, basal kinase activity, and the dynamic behavior of the regulatory domains. Combining biochemical, cellular, and molecular dynamics approaches, we demonstrate distinct regulatory properties for each variant. These findings highlight the critical role of the second N3A motif in modulating the H‐form to B‐form transition during PKA regulation.

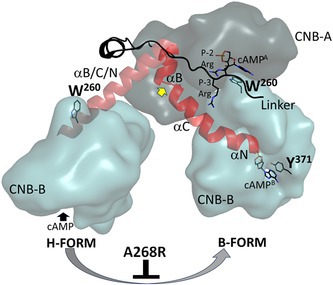

**Abbreviations**
8‐Fluo‐cAMP: 8‐(2‐[fluoresceinyl]aminoethylthio)‐cAMP
BRET: bioluminescence resonance energy transfer
cAMP: cyclic adenosine monophosphate
CNB: cyclic nucleotide binding domain
DD: dimerization/docking domain
FP: fluorescence polarization
Fsk: forskolin
GaMD: Gaussian accelerated molecular dynamics
GFP: green fluorescent protein
IBMX: 3‐isobutyl‐1‐methylxanthine
Iso: isoproterenol
MD: molecular dynamics
PKA: protein kinase A
RIα/RIβ: regulatory subunit type I α/β
RLuc8: Renilla luciferase 8
SPR: surface plasmon resonance

## Introduction

**Fig. 1:**
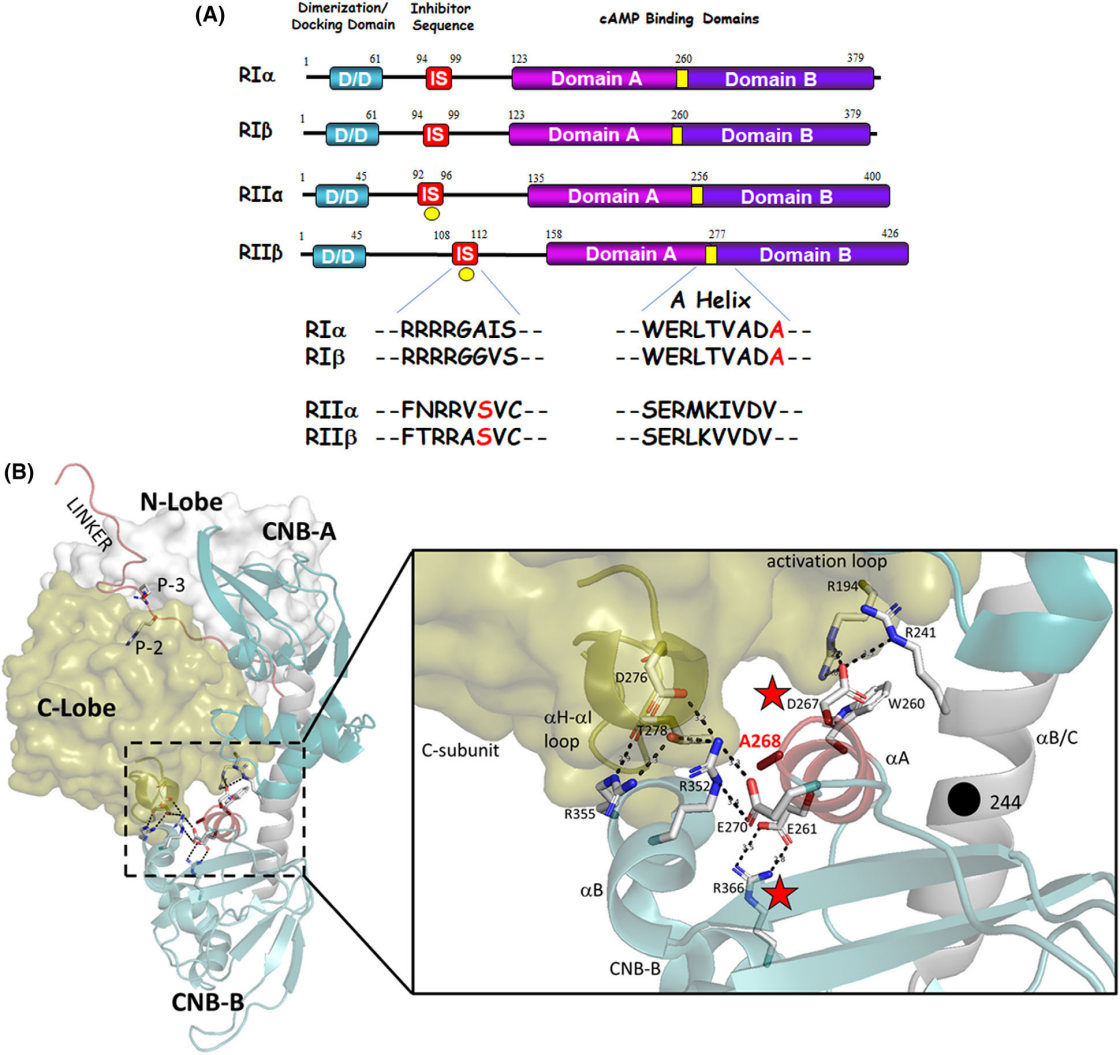
Domain organization of human R subunits and location of the RIβ variation. (A) Comparison of the four regulatory subunits of PKA reveals a conserved domain organization. The RI subunits function as pseudosubstrates, with either an alanine or glycine at the P0 position, while the RII subunits contain a serine. Highlighted in yellow is the A helix of the second N3A motif, at the end of which the RIβ variation is located. (B) The holoenzyme composed of Cα and RIβ (4DIN) highlights position 268 which is altered in the RIβ variants. The red stars indicate the interaction of the second N3A motif with the CNB‐B domain (R366) and the activation loop of the C subunit. D267 plays a crucial role in the allosteric communication between CNB‐A and CNB‐B. Structural model rendered in PyMOL v2.5.

While evolution has provided different mechanisms for regulating the activity of cAMP‐dependent protein kinase (PKA), the main modulation is provided by its regulatory (R) subunits. The four genes encoding the R subunits result in the expression of RI and RII subunits, which each have α and β isoforms (Fig. 1A) [1, 2, 3, 4, 5, 6, 7, 8]. All R subunits share a similar overall architecture that includes a dimerization/docking (DD) domain at the N terminus, followed by an intrinsically disordered region containing the inhibitory sequence (IS) and two cAMP binding domains. While the RII subunits get phosphorylated at a serine located in the IS, RI subunits are pseudo‐substrates, where an alanine or glycine residue in RIα and RIβ, respectively, replaces this serine (Fig. 1A). Despite their high structural similarities, the R subunits are not functionally redundant and display different tissue‐specific localization. While RIα and RIIα were found to be expressed ubiquitously, RIβ and RIIβ subunits are expressed in a tissue‐specific manner [1, 7, 8]. Although both β isoforms are highly expressed in brain, they localize to different areas [9]. RIβ is mainly found in the hippocampus and RIIβ is enriched in the striatum. In addition, RIβ enters the nucleus, while RIIβ is excluded from the nucleus [9]. Furthermore, not only tissue‐specific but also cell‐type and subcellular differences have been reported [10]. Early work showed that in rat and mouse brain, RIβ labeling is restricted to distinct neuronal populations such as Purkinje cells, olfactory mitral cells, lateral thalamic neurons, and superior olivary complex neurons, whereas RIα has a broader distribution in brain regions associated with visceroemotional control [11]. Early studies revealed different phenotypes for the knockout of RIβ and RIIβ. In mice, knockout of RIIβ resulted in alterations in motor behavior and loss of neuronal gene expression regulated by PKA [12]. RIβ knockouts, in contrast, displayed deficits in long‐term depression (LTD) and long‐term potentiation (LTP) [13]. The hippocampal synaptic plasticity tuning was not rescued by upregulated RIα expression, and those functional defects in learning are consistent with the localization of RIβ in the hippocampus.

In comparison with the other PKA regulatory subunits, RIβ is poorly investigated and often equated with RIα despite the different localization and distinct phenotypes in knockout studies. We, therefore, sought to carry out a comprehensive biochemical characterization of RIβ. The primary sequence of the human RIβ protein was initially elucidated in 1991, revealing notable disparities in comparison with the mouse RIβ sequence [2]. Subsequent research corrected some of these differences [14]. Notably, a particular variation remained unresolved: the amino acid composition at Position 268 (Human RIβ consists of 380 amino acids, which would require labeling as 270 instead of 268. However, we have decided to follow the nomenclature of bovine RIα in this text, as it is the common labeling in current literature to avoid confusion) (Fig. 1B). In mouse and rat RIβ, this position was reported as alanine; however, the human sequence displayed an arginine at this position. Surprisingly, the human RIβ sequence as annotated in the UniProt database indicates an alanine residue at Position 268, like the mouse and rat proteins (UniProt: P31321). Since previously published RIβ data in the literature have not distinguished between these two variants, and because it is still not clear if the original sequence is an individual variant or due to sequencing error, we looked more closely at this region of the RIβ structure and found that it is part of a potentially important site that had not been previously well‐characterized.

Residue 268 is located at the beginning of the second cyclic nucleotide binding domain (CNB‐B, Fig. 1B) and is part of a helix‐turn‐helix motif called the N3A motif [15]. Much is already known about the first N3A motif (N3A^A^) located at the beginning of CNB‐A and the importance of this motif in maintaining the dimeric state of the RI subunits [16, 17]. It is an allosteric hotspot for communication between the CNB‐A domain and the CNB‐B domain and for linking cAMP binding to the R‐subunits to allosteric activation of the C‐subunits [18]. Several Carney complex diseases (CNC) and acrodysostosis‐1 (ACRDYS) mutations in RIα are also located in or near these motifs [16]. Finally, N3A^B^ is also the critical ‘hinge point’ between the CNB‐A and CNB‐B domains. In this study, we performed a comprehensive characterization of both RIβ variants, A268 and R268, and found significant differences that highlight the importance of N3A^B^ for proper RIβ‐subunit function and its involvement in the communication of CNB‐A and CNB‐B.

## Results

### RIβ R268 has a reduced cAMP sensitivity

**Fig. 2:**
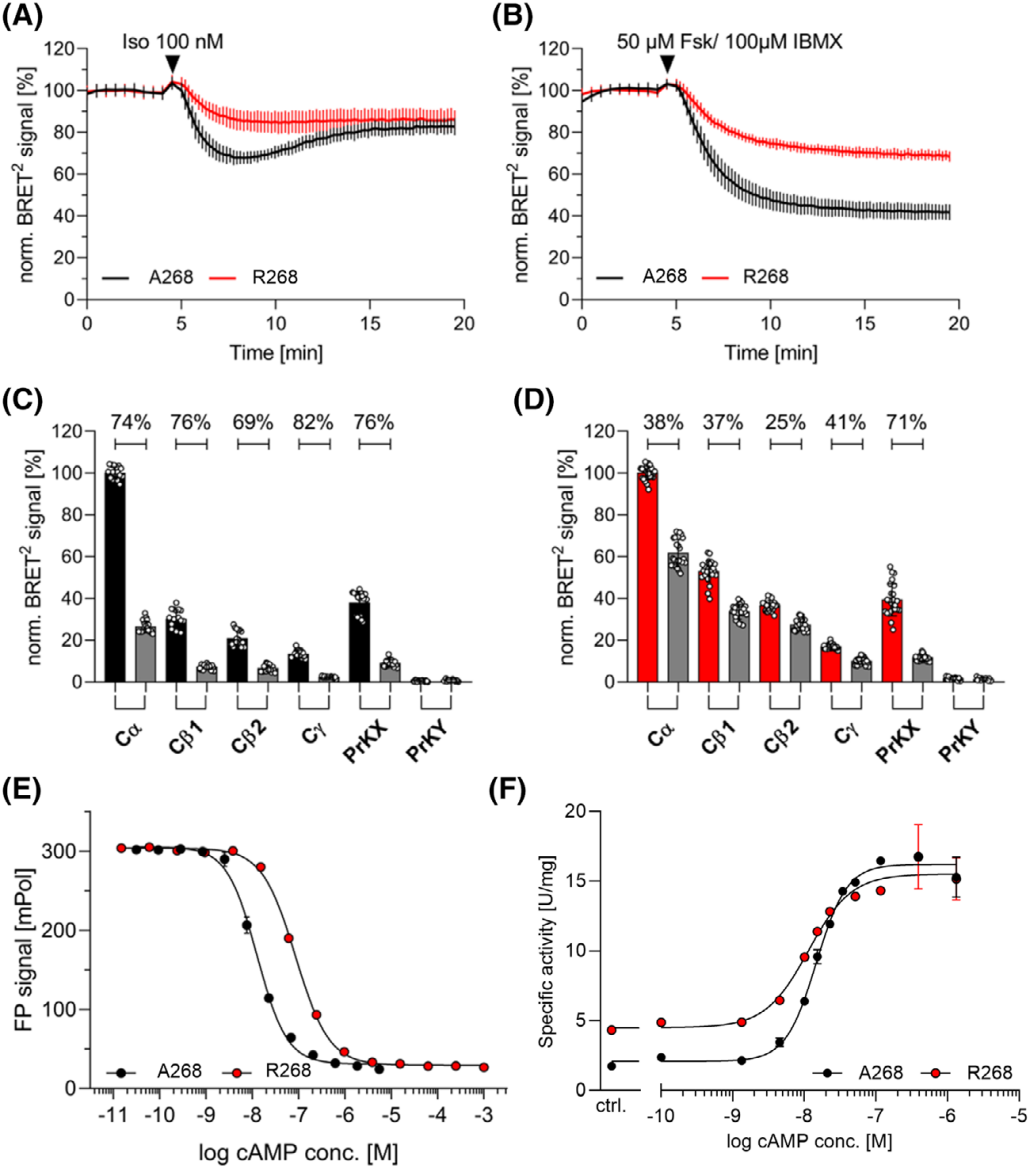
RIβ variants show distinct cAMP‐induced dissociation, affinity and activation. (A) Time dependent dissociation of PKA holoenzymes composed of Cα and one of the respective RIβ variants was induced by 100 nm Iso after 5 min. (B) Stimulus with 50 μm Fsk/100 μm IBMX to maximally increase the cAMP level in cells. (C, D) In endpoint measurements the tendency of a stronger cAMP‐induced dissociation for the A268 variant was confirmed for all tested C subunits, except PrKX. Data were normalized to Cα and the respective RIβ variant. Dissociation was induced with 50 μm Fsk and 100 μm IBMX and measured after 20 min (gray bars). To determine the maximal dissociation (%) a control group (A268: black, R268: red) was treated with buffer and no additional stimulus. (E) cAMP competition assays with 8‐Fluo‐cAMP indicate a reduced cAMP affinity for R268. Fixed concentrations of the respective RIβ variant (2.5 nm) and 8‐Fluo‐cAMP (0.5 nm) were measured with increasing cAMP concentrations. (F) RIβ variants display altered cooperativity. For *In vitro* activation measurements of holoenzymes composed of the RIβ variants kinase activity was measured with a fixed concentration of the holoenzymes (5 nm) and increasing cAMP concentrations. Differences between the RIβ variants were found in particular for the Hill slope as well as for the basal activity.

Comparison of cAMP‐induced dissociation of the PKA holoenzymes in living cells was achieved using a bioluminescence resonance energy transfer (BRET^2^) system. We monitored the dissociation of holoenzymes composed of the RIβ variants and different catalytic subunits in a time‐dependent manner. To increase cAMP concentrations, HEK293 cells were treated with either 50 μm forskolin (Fsk) and 100 μm IBMX (3‐isobutyl‐1‐methylxanthine), or 100 nm isoproterenol (Iso). Fsk/IBMX is considered a very strong stimulus, as the cell‐permeable forskolin directly activates most adenylate cyclases, while the broad‐spectrum phosphodiesterase (PDE) inhibitor IBMX prevents the degradation of cAMP to maintain a high cAMP level. Regardless of the stimulus used, R268 showed a significantly decreased dissociation compared to A268, when measured with Cα (Fig. 2A,B). Moreover, the A268 holoenzyme reassociated a few minutes after the Iso treatment, which was not observed for R268. Measurements with Fsk/IBMX and Cα resulted in a stronger dissociation with no reassociation event within 15 min for both variants. Finally, the maximal dissociation was distinct for both variants. While A268 dissociated up to 60%, R268 only reached a maximum dissociation of approximately 30%. This effect was observed not only for holoenzymes composed of PKA Cα but also for all tested catalytic subunits (Fig. 2C,D, Fig. S1). Only the human protein kinase X (PrKX), a C‐subunit regulated by type I R‐subunits only [19], showed the same percentage of dissociation for both RIβ variants.

Our data suggest that the cAMP affinity is lower for the R variant; however, this is challenging to ascertain in a cellular context. Consequently, we performed fluorescence polarization (FP) measurements to further investigate the cAMP affinity. Both variants exhibited comparable affinities of 4 nm for 8‐Fluo‐cAMP (Fig. S2). However, subsequent FP competition assays using unlabeled cAMP revealed notable differences between the variants (Fig. 2E, Table 1). Due to a decreased cAMP affinity, more unlabeled cAMP is needed to displace 8‐Fluo‐cAMP from R268 (EC_50_: 62 nm) compared to A268 with a 6.8‐fold reduced EC_50_ value (9.1 nm). The decreased cAMP affinity is in line with the BRET^2^ measurements showing reduced cAMP‐induced dissociation in HEK293 cells.

**Table 1:** cAMP competition assays with 8‐Fluo‐cAMP indicate increased EC_50_ values for cAMP for R268. Data are means of at least seven independent measurements from four protein preparations. Unpaired *t*‐test (two‐tailed) was performed indicating significant differences (****). (****P < 0.0001). Analysis was performed using GraphPad Prism 8.0.1.

	RIβ A268	RIβ R268
cAMP (EC_50_)****	9.1 ± 4.3 nm	62 ± 21 nm

### Variations in the second N3A motif alter the cAMP cooperativity

To investigate the holoenzyme activation with cAMP *in vitro*, we determined the activation constants of both variant holoenzymes performed with Cα using a spectrophotometric kinase assay [20]. We found significant differences in the Hill slope as well as for the basal activity of the holoenzyme in the absence of cAMP (Fig. 2F, Fig. S3, Table 2). The difference in cooperativity (1.9 versus 1.6 for A268 and R268, respectively) highlights the importance of the second N3A motif for the communication between the cAMP binding sites. The activation constant (*K*_act_) was slightly higher for A268 (17 nm), indicating a more cAMP‐sensitive holoenzyme when composed with R268 (12 nm) (Fig. 2F, Table 2). These results seem to be in contrast with the BRET measurements in HEK293 cells. Nevertheless, it is important to emphasize that both assays are not directly comparable. First, the conditions under which the spectrophotometric kinase assay was performed are with high magnesium and ATP concentrations, which may differ in the HEK293 cells. Moreover, the spectrophotometric assay determines protein kinase activity with the peptide substrate Kemptide, not the dissociation of the respective holoenzymes. There is still an ongoing discussion if the holoenzyme needs complete dissociation to display kinase activity or not [21, 22, 23]. Additionally, it is conceivable that the higher basal activity observed for the R268 holoenzyme affects the activation constant. To gain more insights into the affinity and stability of the holoenzymes, we performed SPR measurements.

**Table 2:** Activation constants of R variant holoenzymes using cAMP. Data are means of eight measurements from five independent protein preparations and are given with the standard deviation (±SD). To verify the differences unpaired *t*‐tests (two‐tailed) were performed (ns: *P* ≥ 0.05; *P < 0.05; **P < 0.01; ****P < 0.0001). Analysis was performed using GraphPad Prism 8.0.1.

	RIβ A268	RIβ R268
*K*_act_ (nm)*	17 ± 2.2	12 ± 4.6
Hill slope**	1.9 ± 0.2	1.6 ± 0.1
Basal activity (U·mg^−1^)****	1.1 ± 0.6	4.4 ± 1.1
Maximum activity (U·mg^−1^)^ns^	12 ± 3.9	12 ± 5.2

### Both variants display high‐affinity binding to the C‐subunit

**Fig. 3:**
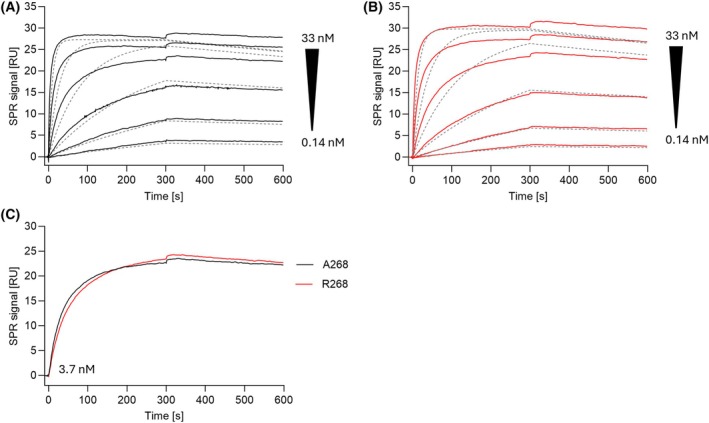
Comparison of the RIβ variant affinities to FSS‐Cα. In a SPR multicycle approach, the FSS‐Cα subunit was immobilized on a dextran matrix and the interaction with different concentrations of the RIβ variants was measured. (A) displays the Cα interaction with A268 (black), and (B) shows the results for R268 (red). The concentration of RIβ ranged from 33 nm to 0.14 nm using a 3‐fold serial dilution series. Both variants display high affinity binding determined with a 1 : 1 Langmuir fit (dashed lines indicate the global fit to the experimental data) provided by the Biacore T200 evaluation software 3.0. (C) To emphasize the similarity of the binding curves of both variants, the measurements with 3.7 nm of the respective RIβ variants were plotted together in one graph.

To determine the affinity for both RIβ variants to the catalytic subunit Cα, SPR measurements were performed immobilizing the C‐subunit via an N‐terminal FSS‐tag to a Biacore sensor chip and analyzing the R variants as analytes (Fig. 3, Table 3). We did not find differences in the affinities of FSS‐Cα to A268 (*K*_D_: 0.11 nm) when compared to the R268 variant (*K*_D_: 0.16 nm). Such high‐affinity bindings are at the detection limit of the T200 Biacore device and must be interpreted with caution. Nevertheless, both variants bound with a high subnanomolar affinity to the C‐subunit, revealing that in the absence of cAMP the affinities are not significantly altered by the variation in the second N3A motif. The similarities are highlighted when respective curves with the same protein concentration (3.7 nm) of the A and R variant were plotted in the same diagram (Fig. 3C). While analyzing the data, we observed that the 1 : 1 Langmuir binding model did not provide a reasonable fit, as it did not capture the association and dissociation phases of the R subunits (analyte) to Cα (ligand). The RIβ subunits are dimers, and it can be assumed that a more complex interaction occurs in our SPR setup. The dimer is thought to bind initially to one immobilized C subunit. The second R subunit is then brought into close proximity to another C subunit, which is bound to the flexible dextran matrix, and interacts with the second C‐subunit. This may be explained by a more complex model (Fig. S4, Table S1). We, therefore, applied a bivalent analyte model of analysis, which yielded highly improved curve fits reflected in lower chi^2^ values for the respective fits. However, the model is speculative and requires further investigation for confirmation.

**Table 3:** Comparison of A268 and R268 affinities to FSS‐Cα using a 1 : 1 Langmuir binding model. Data are generated using a 1 : 1 Langmuir binding fit provided by the Biacore T200 evaluation software 3.0.

	*k*_ass_ (× 10^6^ M^−1^ s^−1^)	*k*_diss_ (× 10^−4^ s^−1^)	*K*_D_ (nm)	Rmax (RU)	Chi^2^ (RU^2^)
RIβ A268	3.1	3.4	0.11	27	1.75
RIβ R268	2.2	3.6	0.16	30	1.69

### Gaussian MD simulations confirm altered dynamics between the variants

**Fig. 4:**
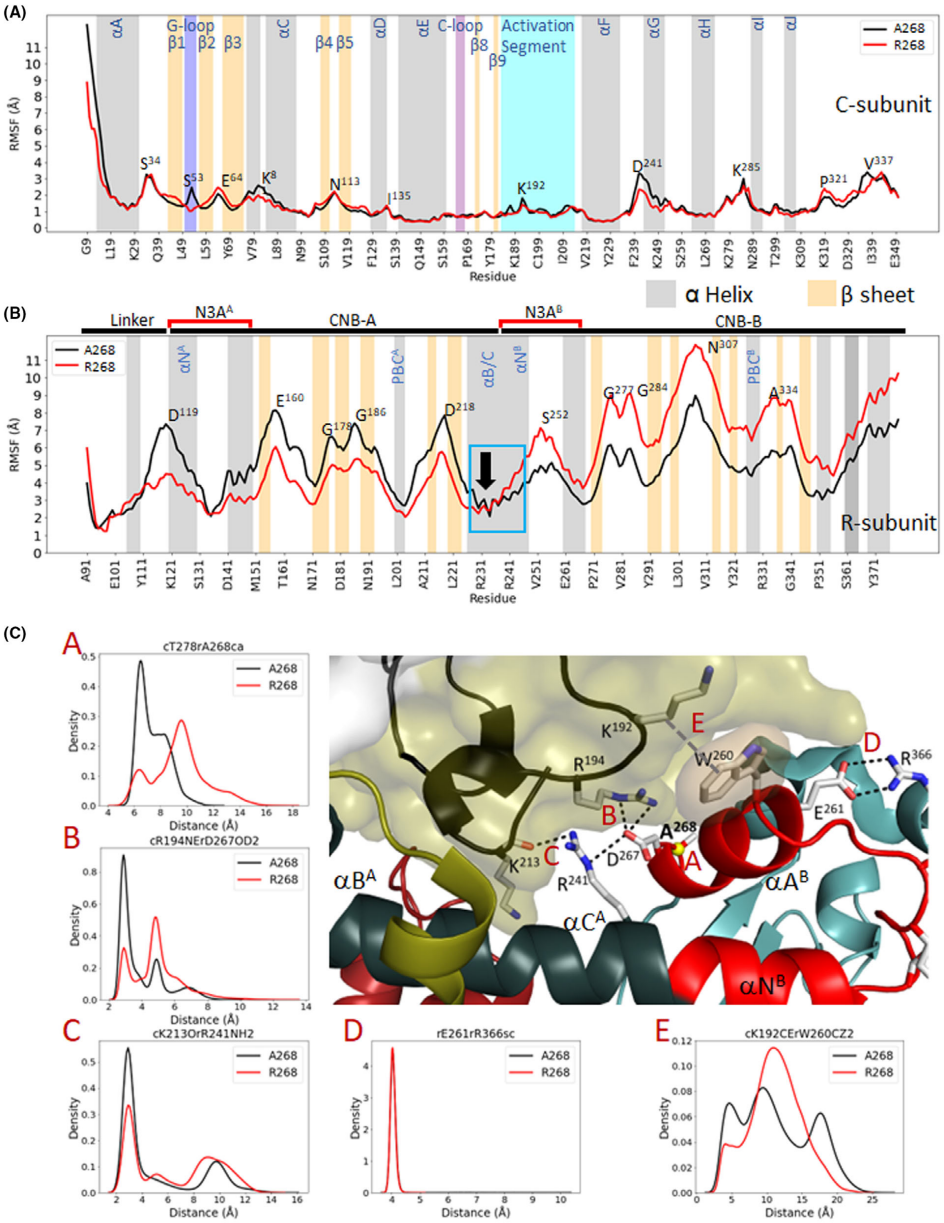
Dynamic differences between A268 and R268 variants of PKA observed in MD simulations. (A) Root‐mean‐square fluctuation (RMSF) plots for the PKA catalytic (C) subunit comparing A268 (black) and R268 variant (red). (B) Root‐mean‐square fluctuation (RMSF) plots for the PKA regulatory (R) subunit comparing A268 (black) and R268 variant (red). Secondary structure elements are indicated: α‐helices in gray and β‐sheets in light orange. Key functional regions, including the Gly‐loop, catalytic loop, and activation segment, are labeled. The activation segment of Cα‐subunit in panel (A), colored in blue, and αB/C helix of R‐subunit in panel (B), in blue box, are in the protein:protein interface. The black arrow also shows the hinge point of the αB/C helix. (C) The RIβ structure showing this Cα‐ and R‐subunit interface (PDB: 4DIN). Residue A268 and nearby residues are highlighted. Residue pairs analyzed in panels A–E are indicated with dashed lines. Distance distribution plots for selected residue pairs, marked as red (A–E), comparing A268 (black) and R268 variant (red), show changes in local dynamics. Structural model rendered in PyMOL v2.5.

To create a dynamic portrait of the RIβ:C complex, we compared 200 ns GaMD simulations for the A268 and R268 proteins. Based on the Root Mean Square Fluctuation plots, aligned with the C‐Lobe of the kinase domain, the R‐ and C‐subunits behave very differently (Fig. 4). The C‐subunit is more stable overall and shows only a few minor differences that correlate mostly with the G‐Loop and the αG Helix where the A268 protein is slightly more flexible (Fig. 4, top panel). In contrast, the RIβ subunit is overall more flexible, and there are significant differences in the dynamic features of the two CNB domains (Fig. 4, middle panel). The CNB‐A domain is more flexible in A268 compared to R268 while the opposite is true for the CNB‐B domain, which is more dynamic in the R268 protein. There is a sharp transition at the B/C/N helix, which is where the two CNB domains are fused. A movie of the simulations shows that the R/C interface is highly dynamic (Movies S1 and S2). Snapshots from those movies showing close‐up views of the R/C interfaces in A268 (left) and R268 (right) are compared to the crystal structure of this interface (middle) in Fig. S5.1.

To delve more deeply into the specific interactions at the R:C interface, we measured specific bond distances of key residues (Fig. 4, bottom panels). We focused first on the αA Helix of CNB‐B (αA^B^) and on residues at the tip of the activation loop. The αA^B^ Helix is a major sensing motif in all PKA R‐subunits; it not only communicates with the C‐subunit but also mediates conversations between the two CNB domains. Most importantly, it is also a sensor for cAMP binding. Two charged residues at the tip of the activation loop of the C‐subunit, R194^C^ and K192^C^, interact directly with key residues in αA^B^. At the C terminus of αA^B^ of RIβ is D267^R^, while W260^R^ is at the beginning of αA^B^. R194^C^ interacts directly with D267^R^ in CNB‐B and R241^R^ in CNB‐A, and these two residues are part of a key allosteric node in all R‐subunits. The charged interactions between R194^C^ and D267^R^ in RIα are different but still close in R268 (Fig. 4C Panel B), and the interactions between D267 and R241 in the αC helix of CNB‐A remain relatively intact (Fig. S5.1). Although the interactions of W260 with the methylene atoms of K192 in the activation loop seem well‐packed in the crystal structure (Fig. S5.1, middle), the MD simulations suggest that the indole ring is quite flexible in A268, and in the R268 protein the interactions are further away and different (Fig. 4C Panel E). In contrast, E261 adjacent to W260 retains close ionic interactions with R366 in the αC' Helix at the end of CNB‐B, similar to the holoenzyme (Fig. 4C Panel D). Collectively, the dynamics show that the R:C interface around this portion of the activation loop becomes more dynamic when A268 is replaced with Arg, while the internal interactions within the CNB‐B domain remain strong supporting the idea that the N3A^B^ motif moves as a rigid body with the core of the CNB‐B domain. Movies show how dynamic this interface is even in the A268 protein and also emphasize how this interface changes as a consequence of replacing A268 with Arg (Movies S1 and S2, Fig. S5.1). The GaMD simulations support the conclusion that the CNB‐B domain becomes dramatically more flexible in R268 while the CNB‐A domain is more flexible in the A268 protein.

Although R241 interacts with the main‐chain carbonyl of K213 in the C‐subunit that follows the conserved APE motif at the end of the P + 1 Loop, in both structures (Fig. 4C Panel C), the entire interface of the αB^B^ helix in CNB‐B with the αH–αI loop in the C‐subunit is completely changed when A268 is replaced with R268 (Fig. S5.2). The rigidity of the CNB‐B domain in R268 is reinforced by the interactions of R370 in the C′ Helix in the CNB‐B domain with the 3_10_ loop in N3A^B^ (Fig. S5.3). Significantly, R370 lies next to Y371, which is the capping residue for cAMP bound to CNB‐B. Y371 as well as R333, which is the phosphate docking site for cAMP in the phosphate binding cassette (PBC), are both solvent exposed and flexible in the holoenzyme.

**Fig. 5:**
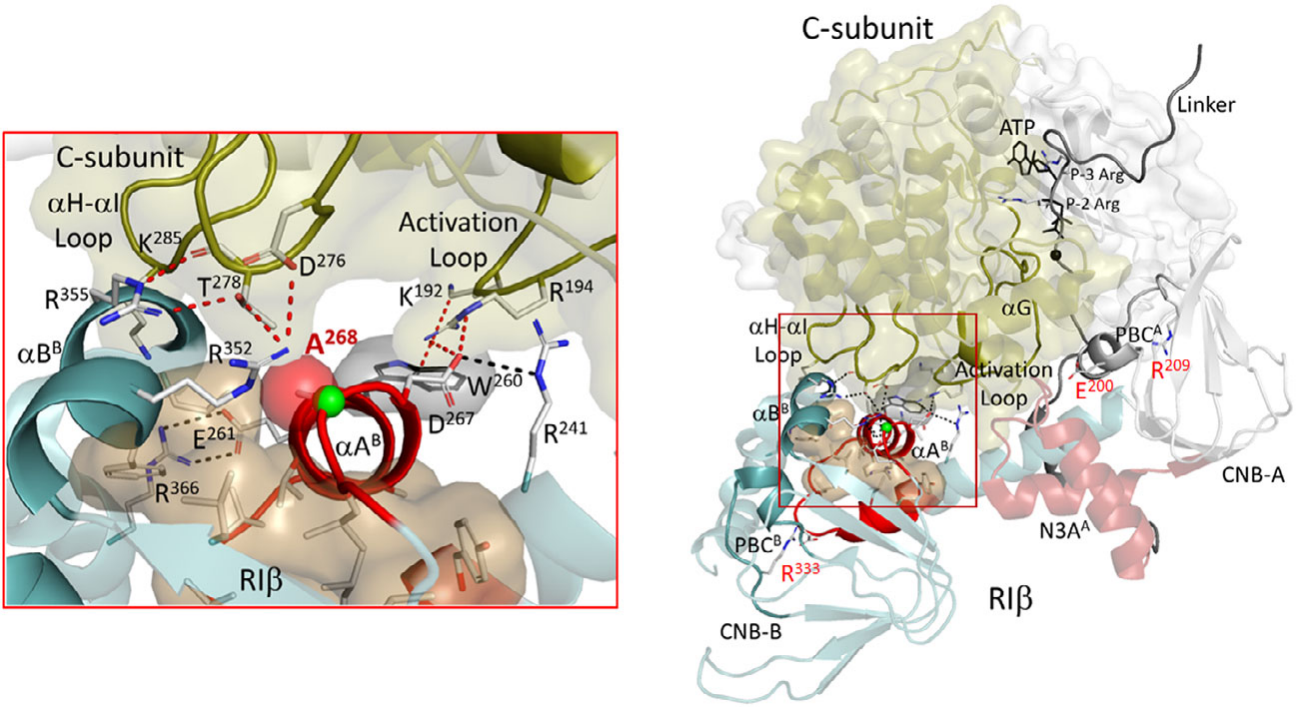
The residue A268 locates in a key position. Right: The RIβ holoenzyme structure showing A268 (green ball) is on the R:C interface between the C‐lobe of the C‐subunit and the CNB‐B domain of RIβ. The N‐ and C‐lobes of the C‐subunit are colored in gray and tan, the linker in black, CNB‐A and CNB‐B in white and light blue, N3A^A^ and N3A^B^ motifs in dark red and red, respectively. A red box highlights the position of A268. Left: A close view of the position of A268. These key residues on the R:C interface were shown. The hydrophobic surface was highlighted by sand shell; A268 is in red shell. The red dashed lines show these interactions were altered in the R268 protein. Structural model rendered in PyMOL v2.5.

**Fig. 6:**
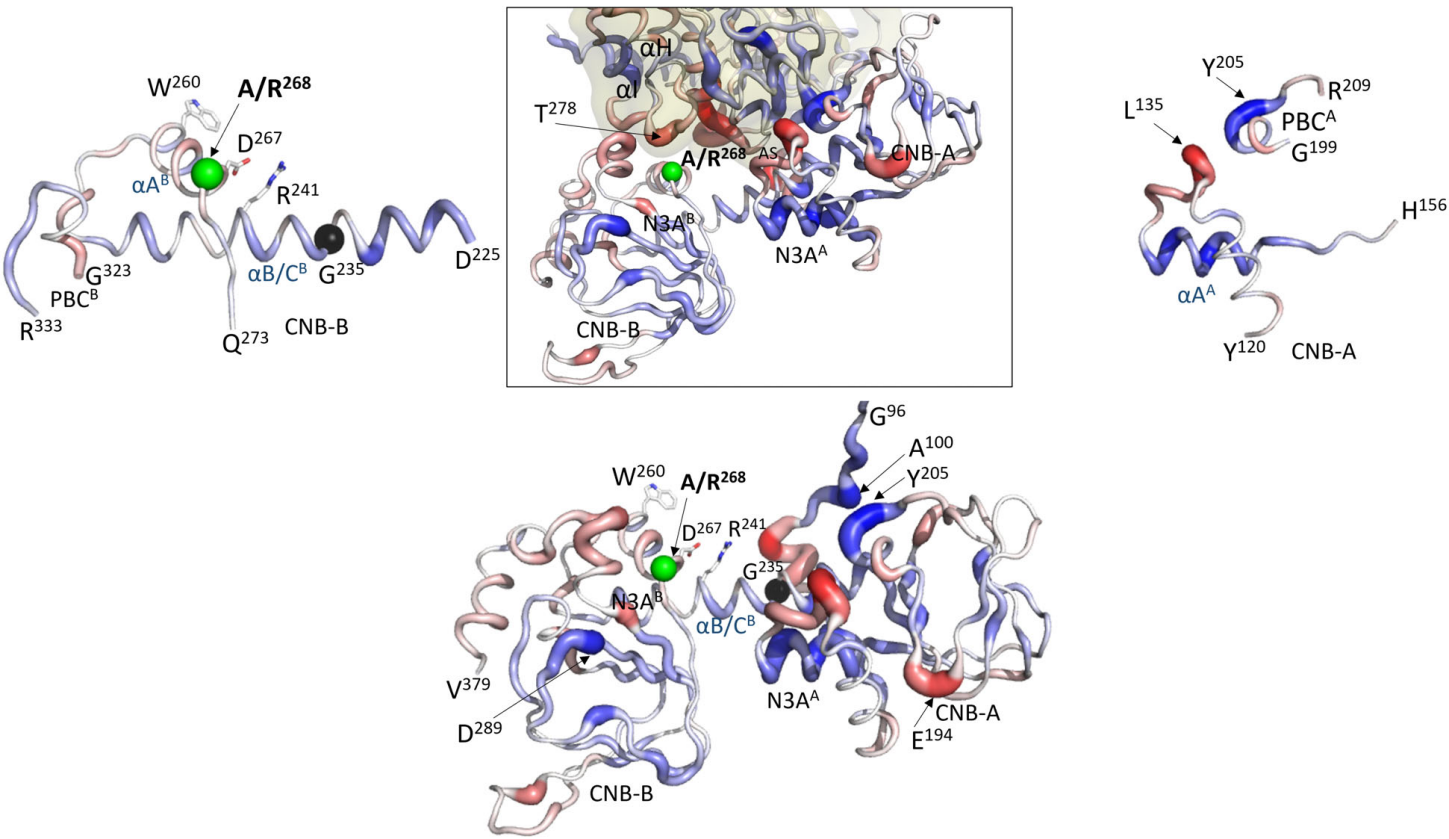
LSP‐based centrality analysis reveals dynamic differences between A268 and R268 variants. Changes in Degree Centrality values between A268 and R268 were mapped onto the crystal structure of the PKA C‐ and R‐subunit complex (PDB ID: 4DIN). Blue regions indicate residues that are more stable (higher centrality) in the R268 variant compared to A268, while red regions indicate residues that are more dynamic (lower centrality) in the R268 variant. Left panels show enlarged views focused on the R‐subunit and the CNB‐B domain. The right panel shows the CNB‐A domain. The bottom panel shows the isolated R‐subunit to better highlight the dynamic changes. Structural model rendered in PyMOL v2.5.

The hydrophobic interactions surrounding A268 and the rest of αA^B^ are substantial and mostly come from the αB^B^ helix and the αN^B^ helix of CNB‐B (Fig. 5). The methyl side chain of A268 also packs against the R/C interface, specifically against the methyl side chain of T278 in the C‐subunit (Fig. 5). Although the packing of this methyl side chain against the C‐subunit is small, it is nevertheless significant, and this interface is broken when A268 is replaced by Arg (Fig. 5, Fig. S6.1). The R268 side chain not only sterically interferes with the R/C interface; it also interferes with the electrostatic interactions (Fig. S5.1). There are two major electrostatic nodes at the R/C interface; one is nucleated by D267 and the other by D276 and T278 in the C‐subunit. R268 toggles between both sites, while in A268 the interactions of D267 are mainly with R241 in RIβ and R194 in the C‐subunit and sometimes with K213 in the C‐subunit. Clearly the interface of the C‐subunit with the αA^B^ Helix is highly dynamic in both proteins, but the communication is dramatically different in R268 (Fig. 6 and Fig. S7, see below).

**Fig. 7:**
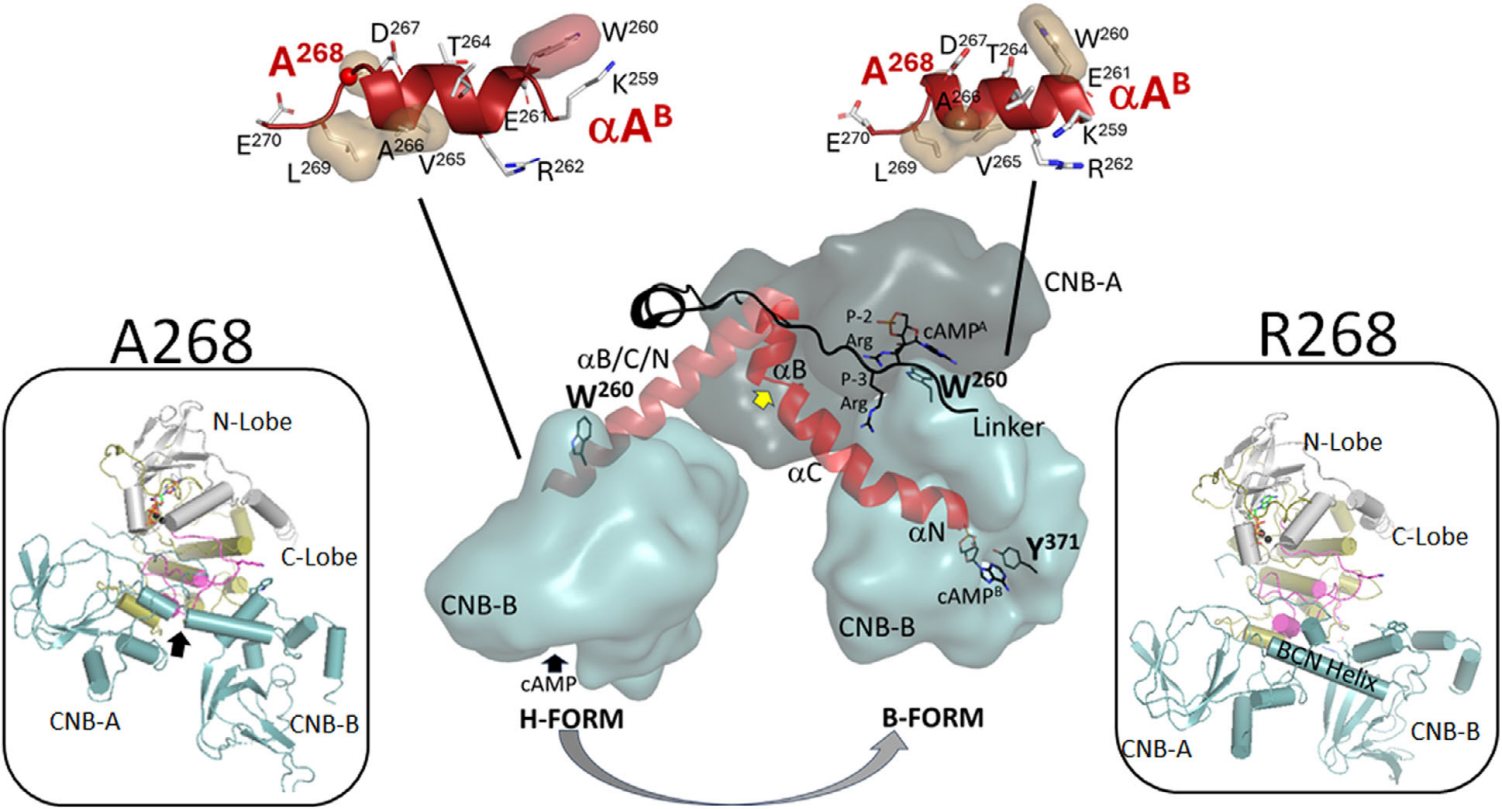
A kink in the BCN helix in A268 but not R268 protein. Top: αA helix (red) of CNB‐B in the RIβ holoenzyme conformation (Left, H‐form) and cAMP bound RIα (Right, B‐form). Center: The BCN helix is extended in H‐form, but bends in B‐form. The yellow arrow shows the bent site, the residue G235. The CNB‐A and CNB‐B domains of RIβ are colored in dark blue and light blue, respectively. The BCN helix is in red. Bottom Left and Right: The snapshots from MD simulations of the A268 (Left) and R268 (Right) proteins. There is a kink at the residue G235 in A268 but not R268 indicated by a black arrow. Structural model rendered in PyMOL v2.5.

A final striking difference between A268 and R268 is the kink between the αB^A^ helix and the αC^A^ helix (Fig. 7 and Fig. S6.2). Bending at this site between the two helices, which is seen in A268, is abolished in R268, and this kink is part of the transition between the (holo) H‐conformation and the (cAMP bound) B‐conformation. The kink, clearly seen in the helix propensity plots (Fig. S6.3), is associated with M234 and G235. The importance of G235 was predicted computationally [24] and has subsequently been validated by SAXS, MD simulations and mutagenesis [18].

To more comprehensively explore the entropic energy contributions to the interactions between the R‐ and C‐subunits, we performed Local Spatial Pattern (LSP)‐based centrality analysis (Fig. 6 and Fig. S7) to quantify localized thermal vibrations from the MD trajectories [25]. By calculating the degree centrality (DC) values for each residue, we assessed how the A268R mutation alters entropic properties across the protein complex. These computational predictions suggest that the A268R substitution leads to widespread changes in entropy‐driven allosteric behavior. The effects are not limited to the mutation site but extend across the entire protein. Many structural motifs in both R‐ and C‐subunits exhibit reduced entropic energy (greater stability), while others become less stable (Fig. 6 and Fig. S7). The R‐subunit, in particular, shows significant changes in DC values. Notably, even though the variation site is on the CNB‐B domain, substantial changes in flexibility are observed in the CNB‐A domain. For example, αN^A^ and the PBC^A^ motif in CNB‐A become more stable in the R variant, while residues near the 3^10^ loop become less stable. In contrast, the N3A^B^ motif and the PBC^B^ motif within CNB‐B show smaller changes between the A and R variants. Additionally, the N3A^A^ linker in the R subunit and the activation segment and αH in the kinase domain show distinct changes in stability and flexibility. While we eventually need to analyze these changes more deeply, our initial comparison demonstrates how a single mutation can exert long‐range entropic effects throughout the protein, altering properties potentially relevant to allosteric signaling (Fig. S7).

### Cellular localization of the RIβ variants

**Fig. 8:**
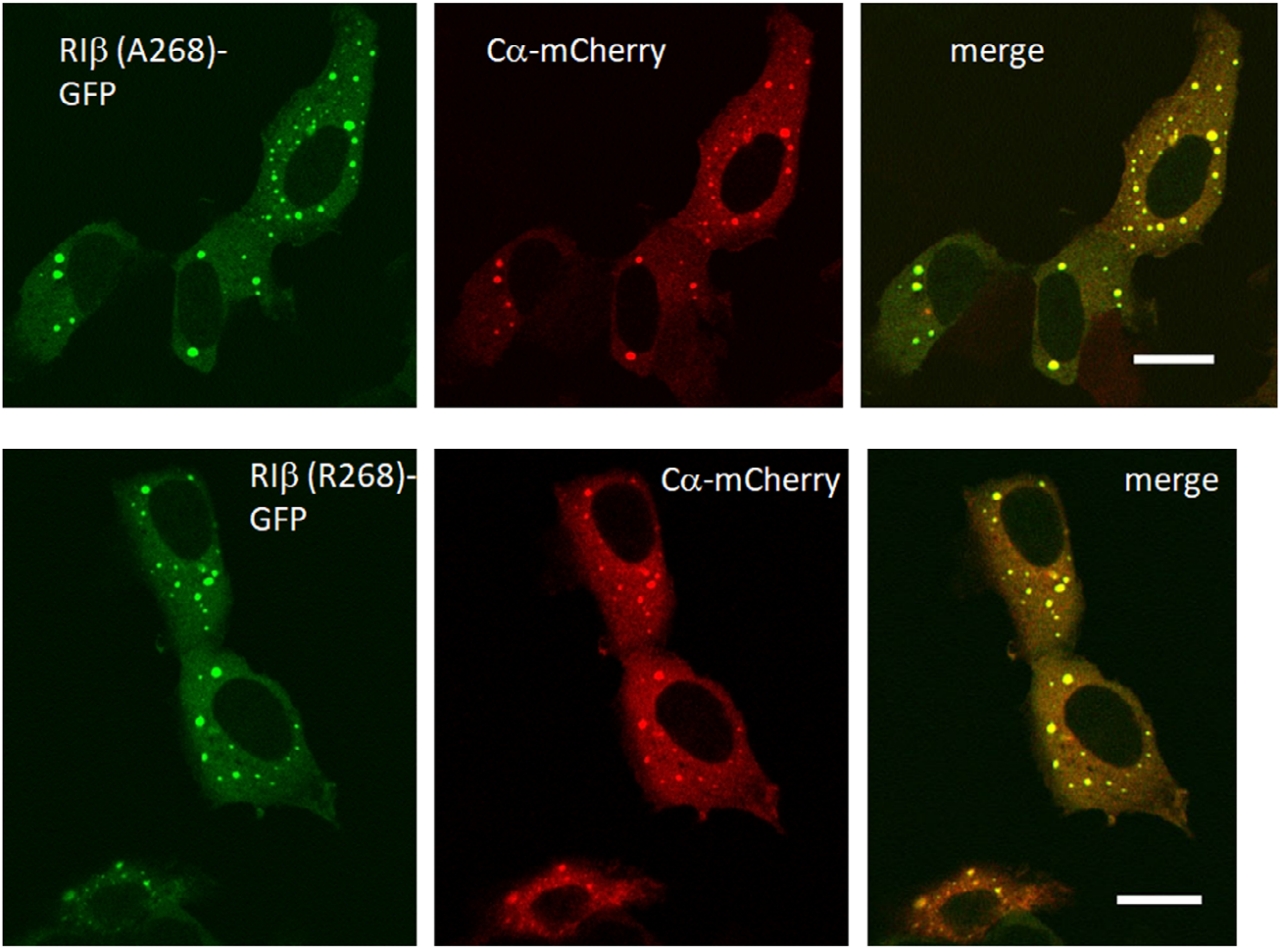
Co‐expression of hRIβ‐GFP and mCα‐mCherry in HEK293a cells. Human RIβ was tagged with GFP and mouse Cα was tagged with mCherry were co‐transfected in HEK293a cells for 16 h before fixation with 4% formaldehyde in PBS. The localization of these two proteins in cells was detected with confocal microscopy. Nuclei were stained with freshly diluted DAPI at 0.1 mg·mL^−1^ in PBS. These panels contain representative images selected from a total of 5 samples in 2 independent experiments. The representative scale bar (white) indicates 20 μm.

RIα has been shown to undergo liquid–liquid phase separation (LLPS), resulting in the formation of puncta‐like structures that are enriched with cAMP and the PKA‐C subunit [26]. As a final comparison of the A268 and R268 proteins, we carried out imaging in HEK293a cells to determine their cellular localization and assess whether both proteins undergo LLPS. To directly compare the localization of the RIβ variants, we specifically overexpressed A268 and R268 in HEK293a cells (Fig. 8). We note that HEK293a cells harbor genomic alterations, including hypotriploidy, multiple copies of chromosome 17, and adenoviral genome insertions on chromosome 19, which could potentially affect the expression of endogenous PKA subunits. While these limitations should be considered, HEK293a cells are widely used for transient overexpression studies due to their high transfectability. Both variants show a similar localization pattern, enriched in the cytoplasm and detectable only at a low level in the nucleus. Moreover, when overexpressing Cα, both RIβ variants colocalized in the cytoplasm and in puncta‐like structures. Like RIα, RIβ undergoes liquid–liquid phase separation, resulting in the formation of puncta‐like structures enriched with cAMP and the C subunit [26, 27]. The puncta observed here require further investigation; however, it is reasonable to assume that LLPS is a shared characteristic of both RI subunits. The observed structures are most consistent with droplet‐like condensates rather than stress‐induced aggregates, in line with previous studies demonstrating liquid–liquid phase separation of type I PKA regulatory subunits [26, 27, 28, 29]. In line with these reports, the puncta in our experiments colocalize with the C‐subunit. Together, these findings argue against the possibility that the observed structures result from ER stress or unfolded protein response and instead indicate that they represent bona fide PKA condensates.

## Discussion

We have demonstrated that the two RIβ variants, A268 and R268, are biochemically distinct and need to be differentiated. Notably, a comparative analysis of mammalian RIβ subunits consistently reveals an alanine residue at Position 268 (Fig. S8). The R268 variant is reported only once in the initial published sequence of human RIβ, while all subsequent studies identify the A268 variant. Additionally, in RIα, only A268 was reported at this point. The inconsistent characterizations of RIβ in existing literature likely stem from the utilization of these two variants in different studies [30, 31, 32, 33, 34]. Therefore, we recommend that future investigations either explicitly identify the variant used or adopt the canonical A268 variant to ensure consistency and comparability across studies.

This residue, 268, is part of the αAB helix in CNB‐B, which plays a critical role in shielding the surface of the C‐lobe that includes the tip of the activation loop. In this way, it protects the C‐subunit from activation by cAMP, where binding of the first cAMP to CNB‐B initiates activation. However, there is also an extended surface on the C‐lobe that binds to a portion of the C‐linker extending from the Inhibitor site to the first N3A motif of the CNB‐A domain. Both CNB‐A and CNB‐B domains contribute to the R:C interface. A268 lies at the C terminus of the αAB helix, while the N terminus harbors another critical residue, W260, which docks onto the surface formed by the activation loop of the C‐subunit in the holoenzyme. Upon cAMP binding to the CNB‐A domain, W260 rotates to serve as a hydrophobic cap for the adenine ring of cAMP (Fig. 5). W260 is specific to the type I R‐subunits, RIα and RIβ. In the crystal structure (PDB ID: 4DIN), W260 at the beginning of the αA helix interacts primarily with the activation loop of the C‐subunit, while A268, although it touches the indole ring of W260, is hydrophobically packed mostly against the αB helix and β strand 8 of the CNB‐B domain, as well as the αH–αI loop in the C‐lobe of the C‐subunit (Fig. 5, inset).

Using biochemical, biophysical, and computational tools, we explored the consequences of replacing the critical residue A268 in the αA helix of CNB‐B with an Arg. The A268R substitution induces significant structural and functional changes in the regulatory subunit of PKA. Although the interactions with the catalytic (C) subunit are altered in our simulations (Fig. 4 and Fig. S5.1), the experimentally determined surface plasmon resonance (SPR) data suggest that binding affinity remains comparable to A268 (Fig. 3)—indicating a shift in the nature, rather than the strength, of the interaction. It should be noted that at such high affinities the Biacore measurements approach the detection limit, so differences may still exist. Nevertheless, both variants clearly interact with the C‐subunit with high (subnanomolar *K*_D_) affinity. In the R268 variant, the interface may now be dominated by R268, which our simulations predict to interact primarily with the αH–αI loop of the C‐subunit instead of the activation loop. This shift implies that the interaction might no longer depend on the presence of Mg^2+^ ions, although further validation is needed to substantiate this prediction. Surprisingly, our simulation revealed that this substitution dramatically alters the dynamic properties of both CNB domains in RIβ. Specifically, the A domain becomes less dynamic, likely because it is no longer tightly anchored to the activation loop of the C‐subunit and the αH–αI loop (Fig. 4 and Fig. S5.1). Computational analysis further predicts that the hydrophobic interactions between CNB‐B and the C‐subunit are lost in the R268 variant, weakening the overall R:C interface. A major outcome of this uncoupling is that W260 is ‘freed’ and can now rotate making it available to cap the adenine ring of cAMP in CNB‐A. This behavior closely resembles that of the W260A mutant described previously [35], in which the coupling between CNB‐B and CNB‐A is also disrupted. The importance of W260 as a critical allosteric residue was also highlighted in MD simulation of RIα [36]. Structurally, the CNB‐B domain remains stable in the R268 variant (Fig. S5.2) but appears to be functionally uncoupled from CNB‐A (Fig. 4 and Fig. S5.2), disrupting the usual intramolecular communication between the two CNB domains within the regulatory subunit. This raises the question of whether cAMP can now bind directly to CNB‐A without the prerequisite of allosteric signaling through CNB‐B. While CNB‐B may still bind cAMP, it seems that this binding does not propagate the conformational changes required for full activation. Instead, CNB‐A may function more independently, allowing the holoenzyme to exist in a partially active or even constitutively active state—a possibility supported by both, the holoenzyme activation measurements *in vitro*, and the forskolin‐induced effects observed in cell‐based assays. The activation measurements revealed increased basal activity for the R268 variant even in the absence of cAMP (Fig. 2F), and BRET‐based dissociation assays showed that the R268 holoenzyme is significantly more resistant to cAMP‐induced dissociation compared to A268 (Fig. 2). The more open conformation of the R268 holoenzyme, indicated by our simulations, likely allows the small peptide substrate Kemptide to access the active site cleft and undergo phosphorylation, even in the presence of the bound R‐subunit. Importantly, our experiments show that the overall affinity for cAMP is reduced in R268, which could be attributed primarily to changes at cAMP binding site A. This observation supports the hypothesis that binding to site A is the critical event for full kinase activation, even though CNB‐B may no longer be necessary for this process.

The predicted behavior of the B/C/N helix may also be relevant. The conformation of this helix could play a role in transmitting activation signals, and its bending at the G235 hinge point appears to be hindered in the R268 protein compared to A268. The C‐linker, which extends from the Inhibitor Site of the R‐subunit, wraps around the extended B/C/N helix; this is a hallmark of the holoenzyme conformation. Both RI subunits (RIα and RIβ) act as pseudosubstrates that dock with high affinity into the active site cleft of the C‐subunit in the presence of ATP and Mg^2+^ (Fig. 5). Under these conditions, the C‐linker becomes tightly clamped onto the C‐lobe of the kinase domain, and the cleft is closed. However, in the R268 variant, the altered interface with the activation loop results in a cleft that remains open, potentially destabilizing an inhibitory clamp. The C‐linker and the A‐helix of the CNB‐B domain still appear to stabilize the extended B/C/N helix that bridges the CNB domains. In the RIα holoenzyme, activation normally begins with cAMP binding to the B‐domain, which frees W260 to rotate and cap the next cAMP at site A. This step relies on a spontaneous bending of the B/C/N helix between the αB and αC helices, which appears to readily occur in the A268 variant, but not in R268. In the latter case, cAMP binding is required to induce the bend, making activation more difficult. However, MD simulations provided valuable insights into conformational dynamics, yet proteins are not rigid in the cellular environment and may adopt additional conformations under physiological conditions. Therefore, our simulations should be regarded as a complementary tool that in some cases support our experimental findings, and in others provide testable predictions for future work.

Interestingly, mutations in the regulatory subunit RIβ of PKA (encoded by *PRKAR1B*) have recently been linked to neurological disorders. For instance, the L50R mutation in the dimerization domain has been shown to cause a neurodegenerative syndrome characterized by cerebellar degeneration and movement disorders, likely due to disruption of holoenzyme assembly and impaired cAMP‐dependent activation [37, 38]. In addition, other *PRKAR1B* variants are associated with neurodevelopmental disorders presenting with autism spectrum features, apraxia, and reduced pain sensitivity, implicating RIβ in neuronal plasticity and sensory processing [39]. Given the structural and functional impact of the R268 mutation described in this study, including weakened coupling between CNB domains, increased basal activity, and reduced cAMP sensitivity, it is plausible that similar alterations in PKA signaling could contribute to pathophysiological mechanisms in the nervous system. Whether such a mutation could occur in patients remains to be determined, but the striking phenotypes of known *PRKAR1B*‐associated disorders underscore the importance of tight regulation within the RIβ‐containing holoenzyme.

## Conclusions

Our study revealed significant differences between the two human RIβ variants, A268 and R268, with A268 being defined here as the canonical form. We emphasize the importance of clarifying the variant used in future studies to ensure consistency. The R268 substitution in RIβ disrupts the structural coupling between CNB domains and weakens the interaction with the catalytic subunit, leading to altered dynamics, reduced cAMP sensitivity, and increased basal activity. These changes suggest a shift toward a constitutively active holoenzyme state and highlight a potential mechanism by which mutations in *PRKAR1B* could contribute to disease, particularly in the nervous system. The insights provide a deeper understanding of the structural and functional dynamics of the RIβ subunit and may also have relevance for the RIα subunit since this site is highly conserved in both isoforms.

## Materials and methods

### Expression and purification of PKA

Expression and purification of the human PKA Cα (UniProt ID: P17612) was performed as described before [40, 41]. The expression was performed in BL21DE3 *E. coli* strains (Agilent Technologies, Santa Clara, CA, USA). Cells were induced with 400 μm IPTG and grown for 16 h before being harvested. The expression of His‐RIβ was performed using TP2000 cells (kind gift of Prof. Choel Kim, BCM, Houston, TX, USA) and purified via Ni^2+^‐NTA agarose (MACHEREY‐NAGEL, Düren, Germany). RIβ R268 was generated by site‐directed mutagenesis.

### Cloning and site‐directed mutagenesis

The RIβ gene (UniProt ID: P31321) was a kind gift from R. Solberg and K. Taskén, Univ. of Oslo, and subcloned at the University of Kassel into the pQTEV and pRluc8‐N1 vectors, respectively, for recombinant protein expression and for BRET measurements in HEK293 (RRID: CVCL_0045) cells. The R268 variant was generated by PCR‐based site‐directed mutagenesis using self‐designed primers (Eurofins Scientific SE, Luxembourg) and verified by Sanger sequencing. Cβ1 (UniProt ID: P22694‐1), Cβ4 (UniProt ID: P22694‐4), Cβ4ab (UniProt ID: P22694‐5) constructs were a kind gift from B. Skålhegg, Univ. of Oslo, and were generated by PCR amplification and subcloning into the pGFP‐C3 vector to produce N‐terminal eGFP fusion proteins. All constructs were verified by sequencing (Microsynth Seqlab GmbH, Göttingen, Germany).

### Bioluminescence resonance energy transfer

To monitor the kinetics of protein:protein interactions in HEK293 (RRID: CVCL_0045) cells bioluminescence resonance energy transfer (BRET^2^) was used. Therefore, the N termini of the respective R subunits were fused to *Renilla* luciferase 8 (RLuc8) while GFP^2^ was fused to the C terminus of the human C subunits used. The time‐dependent measurements, as well as the end‐point measurements, were performed in HEK293 cells as described previously [42]. All measurements were performed at 37 °C in Hanks' Balanced Salt Solution w/o Mg^2+^/Ca^2+^ (HBSS, Biowest, Nuaillé, France; pH 7.4) Evaluation and illustration of the data were done with GraphPad Prism 8.0.1 (GraphPad Software, San Diego, CA, USA). Coelenterazine 400a (DeepBlueC™, Biotium, Fremont, CA, USA) was used as a substrate for the luciferase. As stimuli 100 nm isoproterenol (Iso) or a combination of 50 μm forskolin (Fsk) and 100 μm IBMX were used.

### Fluorescence polarization (FP) measurements

To determine the cAMP interaction with the RIβ variants, FP assays were performed. To measure the binding of 8‐(2‐[fluoresceinyl]aminoethylthio)‐cAMP (8‐Fluo‐cAMP) to the R subunit, a dilution series of RIβ was mixed with the cAMP analogue. In the assay, final concentrations of 0.5 nm 8‐Fluo‐cAMP, 20 mm MOPS (pH 7.4), 150 mm NaCl, 0.005% Chaps, and 1 mm DTT were used. For the competition assays, a fixed concentration of 2.5 nm RIβ and a dilution series of cAMP were added. Measurements were performed in black 384‐well BRAND plates (BRAND) in duplicates with a total reaction volume of 60 μL. Measurements were performed at room temperature at pH 7.4. A CLARIOstar plate reader (BMG Labtech) was used for detection. MARS evaluation software (BMG Labtech) was used for blank subtraction and analysis and presentation of the data were performed using GraphPad Prism 8.0.1.

### Spectrophotometric kinase assay

Determination of the activation constant of the two RIβ holoenzymes was achieved via a spectrophotometric kinase assay [20]. Therefore, holoenzymes were generated by mixing Cα with cAMP‐free RIβ in a 1 : 1.2 ratio. The holoenzymes were dialyzed two times at 4 °C in 1 L dialysis buffer (20 mm MOPS (pH 7.4), 10 mm MgCl_2_, 1 mm ATP, 150 mm NaCl, 0.1 mg·mL^−1^ BSA and 2 mm 2‐mercaptoethanol) for 1 h each. In the assay, 5 nm of the respective holoenzyme was used. A dilution series of cAMP was used to determine the activation constant (*K*_act_) of the holoenzymes. Measurements were performed in a 384‐microwell plate (Microplate, 384 well, PS, F‐ bottom, clear, Greiner Bio‐One, Kremsmuenster, Austria) using a CLARIOstar or POLARstar plate reader (BMG Labtech). As a substrate Kemptide (LRRASLG) was used.

### Surface plasmon resonance (SPR)

Measurements were performed on a Biacore T200 instrument (Cytiva, Marlborough, MA, USA) with a constant flow of 30 μL·min^−1^ and at 25 °C and a data sampling frequency of 10 Hz. Strep‐Tactin XT was covalently bound to a CM5 chip (Cytiva) using the Twin‐Strep‐tag^®^ Capture Kit (IBA Lifesciences, Goettingen, Germany). Immobilization was performed in HBS‐P buffer (10 mm HEPES pH 7.4, 150 mm NaCl, 0.05% P20) according to the Capture Kit instructions. Measurements with FSS‐Cα were performed in FSS running buffer (10 mm HEPES pH 7.4, 150 mm NaCl, 0.05% P20, 10 mm MgCl_2_, 1 mm ATP). The contact time for the capture of the FSS‐Cα constructs was 50 s at a constant flow rate of 10 μL·min^−1^. In multicycle approaches, different concentrations of PKA regulatory subunits were injected at a flow rate of 30 μL·min^−1^. After every cycle, the surface was regenerated three times with 3 M GuHCl for 1 min each at a flow rate of 30 μL·min^−1^ allowing for the removal of the PKA‐C/R complex. Collected data were first double‐referenced before subtracting a reference flow cell.

### Gaussian accelerated molecular dynamics (GaMD) simulation and local spatial pattern (LSP)‐alignment based protein residue networks analysis

The PKA holoenzyme Cα and RIβ model was constructed based on the reported PKA structure (PDB ID: 4DIN), and the R268 variant modeled in PyMOL. The charge states of ionizable residues were adjusted to neutral pH (pH 7), and the models were solvated in a cubic box of TIP4P‐EW water molecules with 150 mm KCl and a 10 Å buffer using LEaP in Amber [43]. Energy minimization, heating, and equilibration were conducted in AMBER16, incorporating parameters from the Bryce AMBER parameter database for phosphoserine and phosphothreonine [44]. The minimization steps included hydrogen‐only, solvent, ligand, sidechain, and all‐atom minimizations. Initial minimization applied harmonic restraints to non‐hydrogen atoms with a 10 Å non‐bonded cut‐off, followed by heating from 100 K to 300 K over 250 ps under constant volume and temperature (NVT) conditions with a 2 fs time step. Langevin dynamics were used to maintain temperature, with a 5.0 kcal/mol/Å^2^ positional restraint on the protein backbone. This was followed by constant pressure equilibration (NPT) at 1 bar, with an 8 Å cutoff for nonbonded interactions and restraints on the protein and peptide for 250 ps. An unrestrained equilibration in NPT was conducted for an additional 300 ps. Subsequently, long‐production simulations were carried out using GaMD on GPU‐enabled AMBER16 to enhance sampling of conformational states [45]. GaMD applies a Gaussian distributed boost to the potential energy surface, facilitating transitions between meta‐stable states with reweighting capabilities. Both dihedral and total potential boosts were used concurrently. After collecting potential statistics for 2 ns, GaMD was applied for another 2 ns with periodic updates of boost parameters. Each GaMD simulation was equilibrated for 10 ns prior to data collection, and output coordinates and energies were saved every 10 ps. For each construct, three independent 200 ns GaMD simulation replicates were performed to achieve accelerated conformational sampling [46]. The LSP alignment and network analysis were conducted using a previously described method [25]. From each simulation, 100 structures were extracted over the first 10 ns at 0.1 ns intervals. An all‐to‐all LSP alignment was performed on each set of 100 structures, generating adjacency matrices that were then averaged for each set. Degree centralities were calculated for these averaged matrices, yielding three centrality values per construct. These values were further averaged, and the differences in degree centralities between variants were separated into positive and negative values. The resulting values were scaled from 0 to 100 and mapped onto the model as B‐factors using PyMOL. Root Mean Square Deviation (RMSD) analysis was performed using backbone atoms only. Structures were first aligned to the αE and αF helices of the C‐subunit. Kernel density estimation (KDE) was used to generate smooth distance distribution plots for analyzing interaction between the RIβ and C‐subunit across the MD trajectories.

### Cell imaging

Human RIβ‐GFP was prepared in pcDNA4‐zeosin (Thermo Fisher, Waltham, MA, USA) and given by Dr. Mandy Diskar. Subsequent mutations were performed based on this construct with PCR. Mouse Cα‐mCherry was subcloned in pcDNA3 (Thermo Fisher) and given by Dr. Brent Martin. The transfections of HEK293a (RRID: CVCL_6910) and HeLa (RRID: CVCL_0030) cells were performed with Polyfect (Qiagen, Hilden, Germany) following the manufacturer's instructions. After expression for 16 h, the cells were fixed with 4% formaldehyde in PBS. The antibodies used in the experiments were sheep anti‐RIβ 1 : 300 dilution (R&D, AF4177), rabbit anti‐Cα 1 : 100 dilution (Taylor lab in‐house polyclonal antibody), and rabbit anti‐Cβ 1 : 100 dilution (LSBio, LS‐C191947) [47]. The color‐conjugated secondary antibodies used were AlexaFluor™ 488 Donkey anti‐Rabbit IgG (A21206; Thermo Fisher), and Cy3 Donkey anti‐Sheep IgG (713165147; Jackson ImmunoResearch, West Grove, PA, USA). The solution to block samples and dilute antibodies consisted of donkey serum 3%, BSA 1%, fish gelatin 1%, Triton X‐100 0.1%, and glycine 50 mm in PBS (pH 7.0). All cell lines were regularly tested and confirmed to be mycoplasma‐free.

## Conflict of interest

The authors declare no conflict of interest.

## Author contributions

M.W., F.W.H., and S.S.T. were involved in conceptualization. F.W.H. and S.S.T. were involved in funding acquisition. M.W., V.P., Y.M., J.‐H.W., and J.W. were involved in investigation. M.W., Y.M., J.‐H.W., and J.W. were involved in methodology. F.W.H. and S.S.T. were involved in project administration and supervision. M.W., Y.M., J.‐H.W., and J.W. were involved in visualization. M.W., J.‐H.W., J.W., F.W.H., and S.S.T. were involved in writing – original draft. M.W., V.P., Y.M., J.‐H.W., J.W., F.W.H., and S.S.T. were involved in writing – review and editing. All authors have read and agreed to the published version of the manuscript.

## Supporting information


**Fig. S1.** RIβ variants show comparable cAMP‐induced holoenzyme dissociation for Cβ splice variants.
**Fig. S2.** Respective curves of 8‐Fluo‐cAMP binding to the RIβ variants.
**Fig. S3.** Comparison of basal and maximum holoenzyme activity of the RIβ variants.
**Fig. S4.** Comparison of the RIβ variant affinities to FSS‐Cα analyzed using a bivalent analyte model.
**Fig. S5.1.** Structural comparison of the A268 and R268 variant highlighting interface interactions.
**Fig. S5.2.** R:C interface between αH‐αI loop and αB helix.
**Fig. S5.3.** CNB‐B domain in RIβ:C structure.
**Fig. S6.1.** The residue A268 is on the R:C interface.
**Fig. S6.2.** αA helix of CNB‐B is always on the domain interface.
**Fig. S6.3.** Helical propensity and conformational changes in the B/C helix of the R‐subunit. Left and Right.
**Fig. S7.** Changes in degree centrality between A268 and R268 variant.
**Fig. S8.** Sequence alignment of mammalian RIβ reveals A268 as the canonical variant.
**Table S1.** Association and dissociation rate constants for both the RIβ variant and FSS‐Cα.

**Movie S1.** Snapshots from MD simulations of the A268 variant, showing representative conformations and differences in interactions between the R and C subunits. Hydrogen bonds are shown as dashed red lines.
**Movie S2.** Snapshots from MD simulations of the R268 variant, showing representative conformations and differences in interactions between the R and C subunits. Hydrogen bonds are shown as dashed red lines.

## Data Availability

The datasets generated and analyzed during the current study are available from the corresponding authors upon reasonable request.
