# Supplementary material for: Functional divergence of protein kinase A regulatory subunit Iβ variants: the importance of N3A motifs in PKA regulation
Source: FEBS J. 2025 Dec 13;293(8):2417–34. doi: 10.1111/febs.70358 (PMC13080237; doi:10.1111/febs.70358)
Supplement: Supplementary file 1 — Fig. S1. RIβ variants show comparable cAMP‐induced holoenzyme dissociation for Cβ splice variants. Fig. S2. Respective curves of 8‐Fluo‐cAMP binding to the RIβ variants. Fig. S3. Comparison of basal and maximum holoenzyme activity of the RIβ variants. Fig. S4. Comparison of the RIβ variant affinities to FSS‐Cα analyzed using a bivalent analyte model. Fig. S5.1. Structural comparison of the A268 and R268 variant highlighting interface interactions. Fig. S5.2. R:C interface between αH‐αI loop and αB helix. Fig. S5.3. CNB‐B domain in RIβ:C structure. Fig. S6.1. The residue A268 is on the R:C interface. Fig. S6.2. αA helix of CNB‐B is always on the domain interface. Fig. S6.3. Helical propensity and conformational changes in the B/C helix of the R‐subunit. Left and Right. Fig. S7. Changes in degree centrality between A268 and R268 variant. Fig. S8. Sequence alignment of mammalian RIβ reveals A268 as the canonical variant. Table S1. Association and dissociation rate constants for both the RIβ variant and FSS‐Cα. [file FEBS-293-2417-s002.zip › Functional divergence of Protein Kinase A regulatory subunit.pdf]

Functional divergence of Protein Kinase A regulatory subunit Iβ variants: The importance of N3A motifs in PKA regulation

Maximilian Wallbott[1], Jui-Hung Weng[2], Valeria Pane[1], Yuliang Ma[3], Jian Wu[2, 3], Susan S. Taylor[2, 3, *], Friedrich W. Herberg[1, *]

Affiliations:

[1] Department of Biochemistry, Institute for Biology, University of Kassel, Kassel, 34132, Germany

[2] Department of Pharmacology, University of California, San Diego, La Jolla, CA, U.S.A.

[3] Department of Biochemistry and Molecular Biophysics, University of California, San Diego, La Jolla, CA, U.S.A.

[*] Corresponding Authors

Correspondence: Prof. Friedrich W. Herberg, Department of Biochemistry, Institute for Biology, University of Kassel, Heinrich-Plett-Str. 40, 34132 Kassel, Germany. E-mail: herberg@uni-kassel.de; Tel.: +49 561 804-4511

Correspondence: Prof. Susan S. Taylor, Department of Pharmacology, University of California San Diego, 9500 Gilman Drive, La Jolla, CA 92093, USA. E-mail: staylor@ucsd.edu; Tel.: +1 858 534-3677

Supplementary

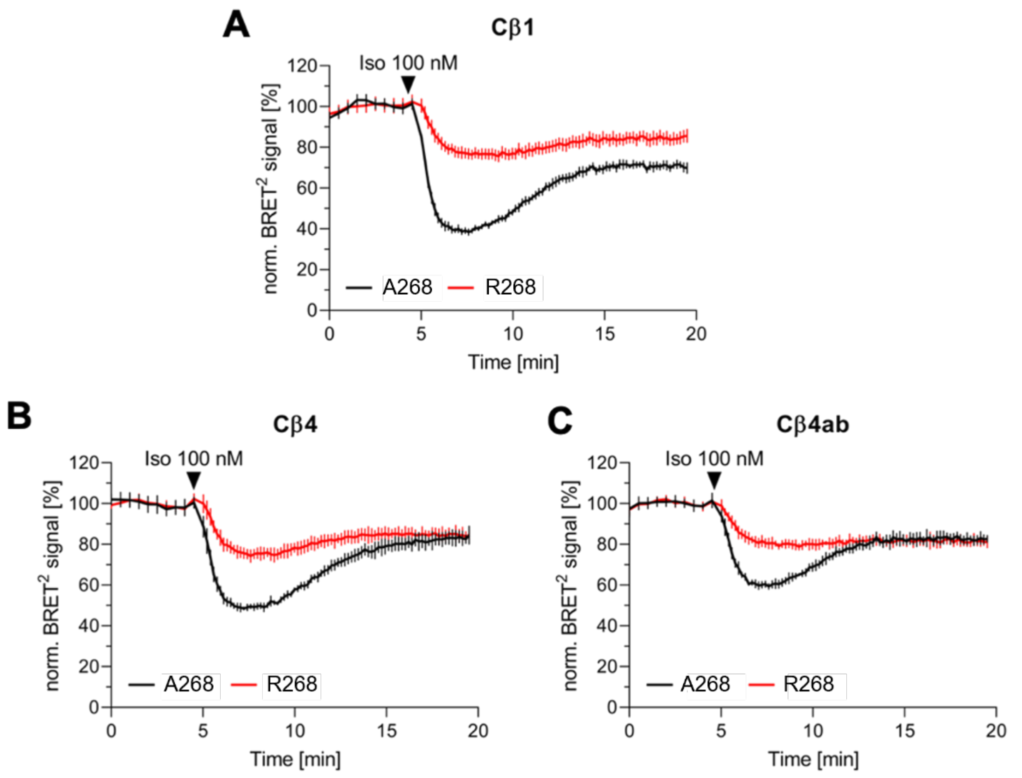

Figure S1: RIβ variants show comparable cAMP induced holoenzyme dissociation for Cβ splice variants. Time dependent dissociation of PKA holoenzymes composed of Cβ1 **(A)**, Cβ4 **(B)** and Cβ4ab **(C)** and the respective RIβ variants was induced by 100 nM Iso after 5 min. Error bars represent six replicates (n = 6) with standard deviation (SD) indicated. Graphs were created in GraphPad Prism 8.0.1.

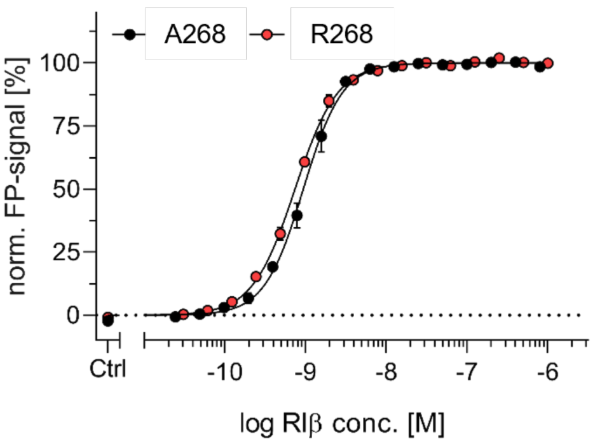

Figure S2: Respective curves of 8-Fluo-cAMP binding to the RIβ variants. The interaction of 8-Fluo-cAMP with the respective RIβ variants were determined in FP-assays. Therefore 0.5 nM of the analog was mixed with a dilution series of the respective RIβ variant. Both variants displayed equal affinities (4.1 nM). Data were normalized by setting the bottom of the respective sigmoidal fit to 0 % and the top to 100 %. Error bars represent two replicates (n = 2) with standard deviation (SD) indicated. The image was created and the analysis was performed using GraphPad Prism 8.0.1.

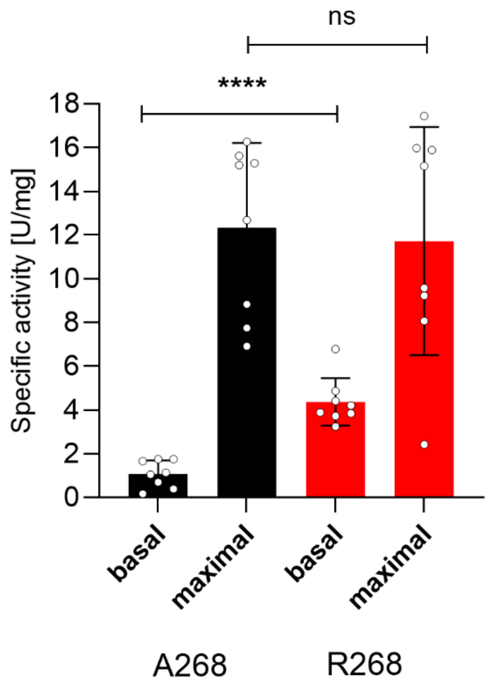

Figure S3: Comparison of basal and maximum holoenzyme activity of the RIβ variants. The basal and maximum activities of a sigmoidal dose-response fits with variable slopes were compared for both variants (respective curves are shown in Fig. 2F). Data are means of eight measurements (n=8) from five independent protein preparations. Error bars indicate the standard deviation (SD). Unpaired t-tests (two-tailed) were performed for both basal and maximum activity. ns: $P \geq 0.05$; *: $P < 0.05$; **:$P < 0.01$;

***: P < 0.001; ****: P < 0.0001. The image was created and the analysis was performed using GraphPad Prism 8.0.1.

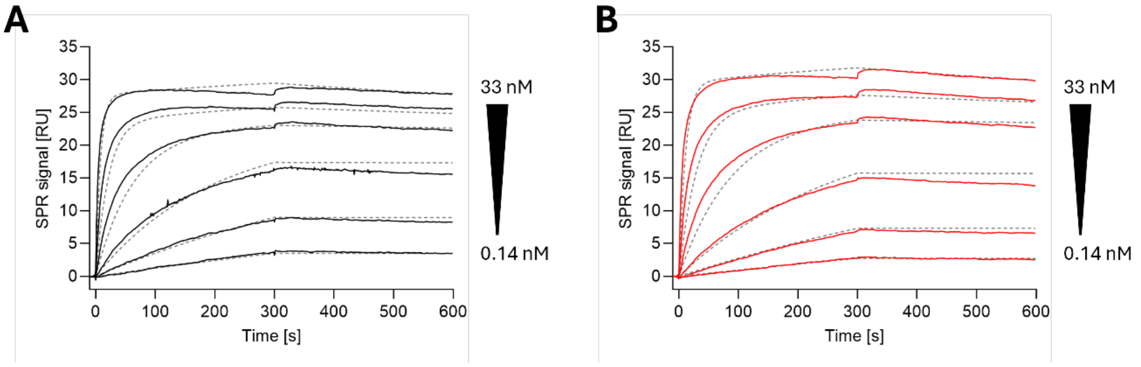

Figure S4: Comparison of the RIβ variant affinities to FSS-Cα analyzed using a bivalent analyte model. In a SPR multicycle approach, the FSS-Cα subunit was immobilized on a dextran matrix and the interaction with different concentrations of the RIβ variants were measured. **(A)** displays the Cα interaction with A268 (black), and **(B)** shows the results for R268 (red). The concentration of RIβ ranged from 33 nM to 0.14 nM using a 3-fold serial dilution series. Both Variants show high affinity binding determined by a bivalent analyte fit provided by the Biacore T200 evaluation software 3.0.

Table S1: Association and dissociation rate constants for both the RIβ variant and FSS-Cα. Data are generated using a bivalent analyte fit provided by the Biacore T200 evaluation software 3.0.

	k_{ass}**1** (1/Ms)	k_{diss}**1** (1/s)	k_{ass}**2** (1/RUs)	k_{diss}**2** (1/s)	**R**$_{max}$ (RU)	**Chi**^{2} (RU^2)
**A268**	1.07E+06	4.22E-04	0.0051	6.02E-04	44	0.659
**R268**	7.61E+05	4.23E-04	0.0034	6.92E-04	47	0.706

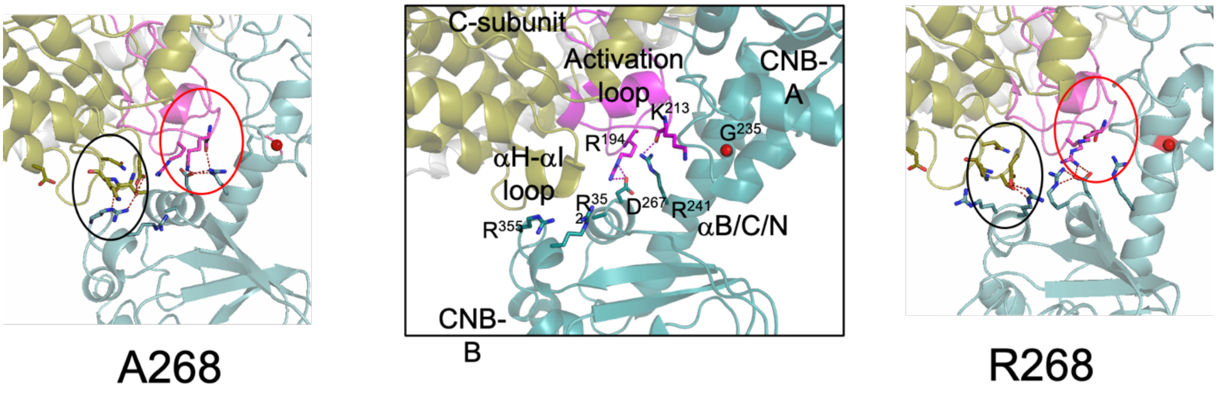

Figure S5.1: Structural comparison of the A268 and R268 variant highlighting interface interactions. Center: Crystal structure of the PKA C-subunit (yellow) in complex with the R-subunit (cyan), based on PDB entry 4DIN. The activation loop is shown in magenta, and key interface residues are labeled. Residue G235 on the B/C helix is marked with a red sphere. **Left and Right:** Snapshots from MD simulations of the A268 (left) and R268 variant (right), showing representative conformations

and differences in interactions between the R and C subunits. Hydrogen bonds are shown as dashed red lines. Structural model rendered in PyMOL v2.5.

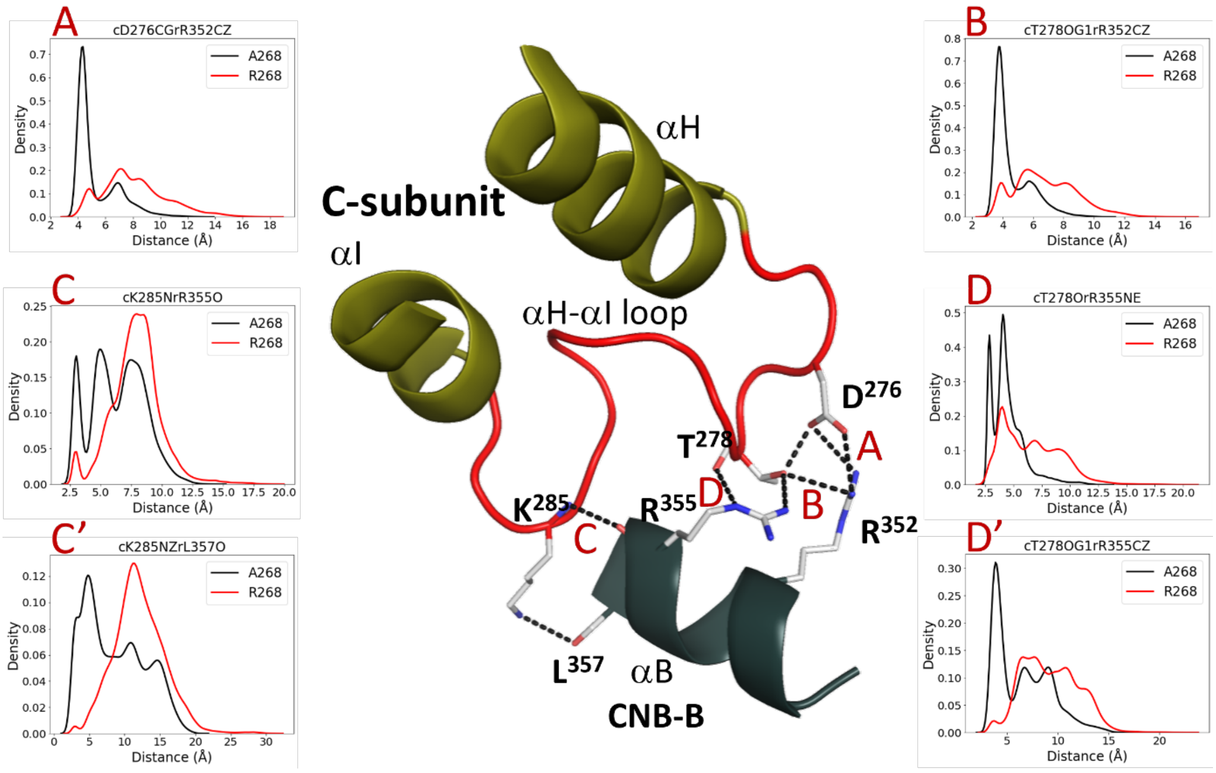

Figure S5.2: R:C interface between αH-αI loop and αB helix. Center: In Rβ structure, αH-αI loop of C-subunit (tan) forms an interface with αB helix of RIβ (dark blue). These interacting residues are shown, and H-bonds are in black dash lines. **Panels A–D':** Distance distribution plots for these residue pairs comparing A268 (black) and R268 (red) variant. Structural model rendered in PyMOL v2.5.

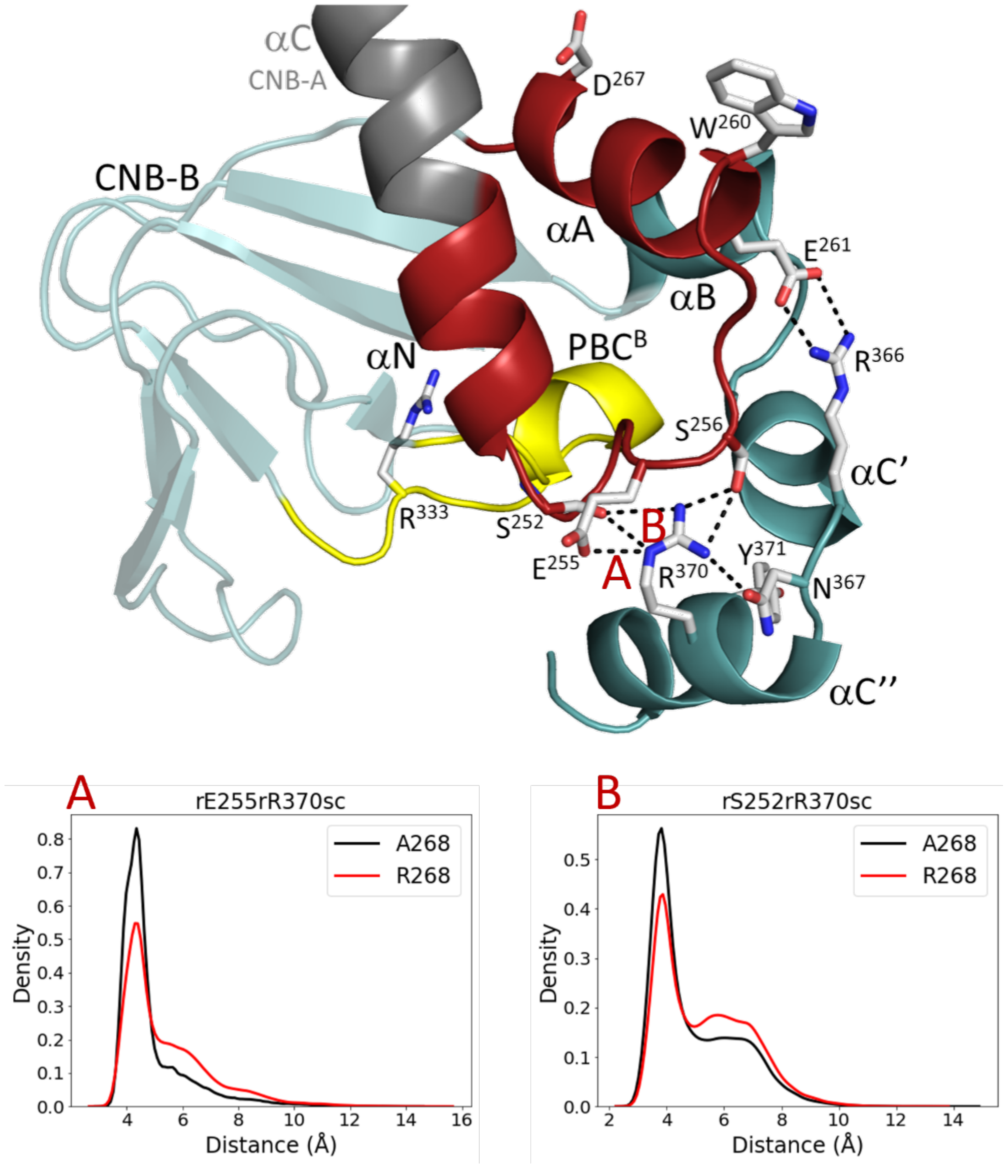

Figure S5.3: CNB-B domain in RIβ:C structure. Top: In RIβ structure, R366 from C'-helix and R370 from C''-helix interact with E261 and several N3A residues, respectively. **Panels A–C:** Distance distribution plots for these residue pairs comparing A268 (black) and R268 (red) variant. Structural model rendered in PyMOL v2.5.

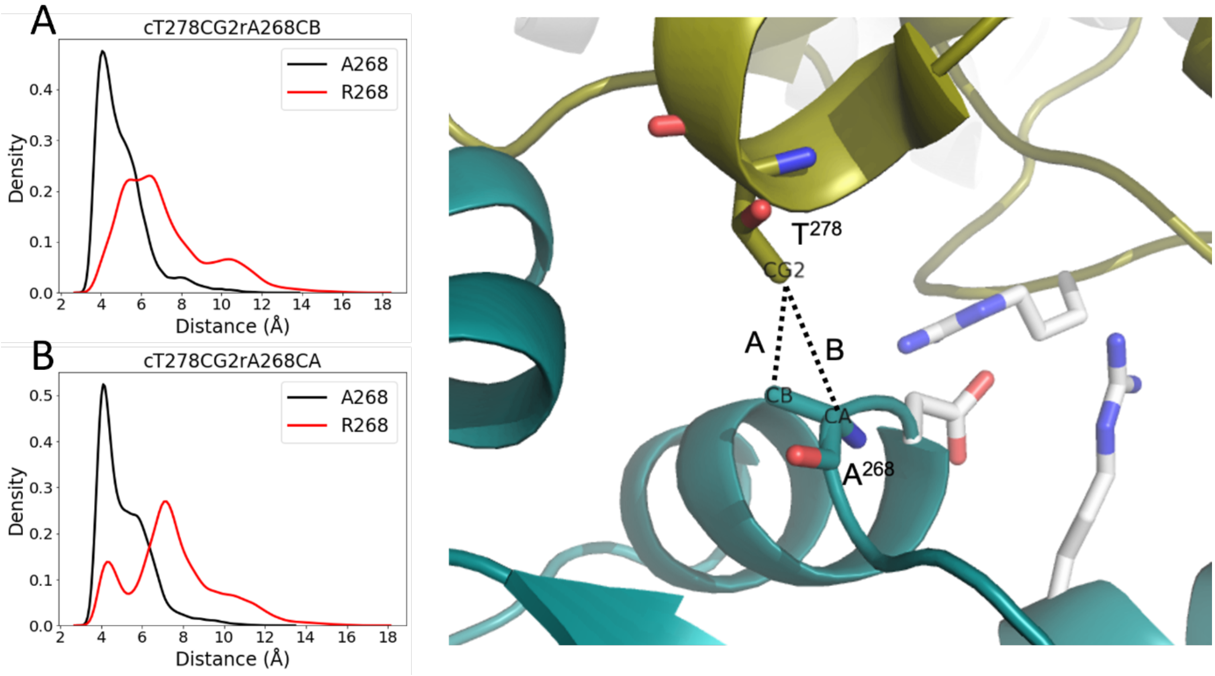

Figure S6.1: The residue A268 is on the R:C interface. Right: A268 of RIβ (cyan) is in a close position to T278 from αH-αI loop of C-subunit (tan). **Panels A–B:** Distance distribution plots for the two residues comparing A268 (black) and R268 (red) variant. Structural model rendered in PyMOL v2.5.

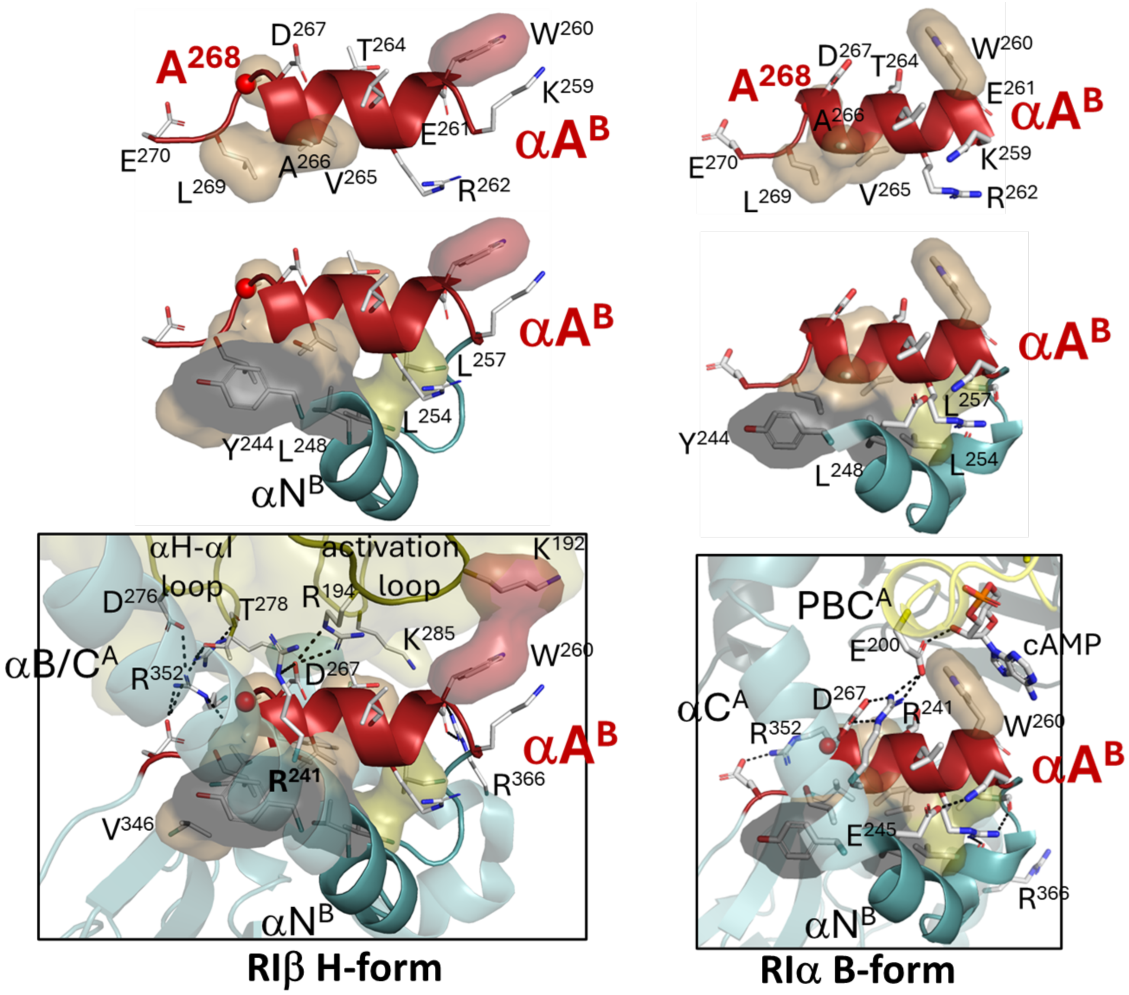

Figure S6.2: αA helix of CNB-B is always on the domain interface. Left top: αA helix (red) of CNB-B in the RIβ holoenzyme conformation (H-form). A268 is labelled with red ball. W260 is shown in a red shell. **Left center:** αA helix forms a hydrophobic pocket with αN helix (cyan) of CNB-B. **Left bottom:** αA helix is on R:C interface in the RIβ holoenzyme. The PKA-C is color in tan, and some key residues from the activation loop and αH-αI loop are highlighted. **Right top:** αA helix (red) of CNB-B in the cAMP bound RIα conformation (B-form). W260 is shown in a sand shell. **Right center:** αA helix forms a hydrophobic pocket with αN helix (cyan) of CNB-B. **Right bottom:** αA helix is on the interface between CNB-A and CNB-B domains in the B-form. Some key residues are highlighted. Structural model rendered in PyMOL v2.5.

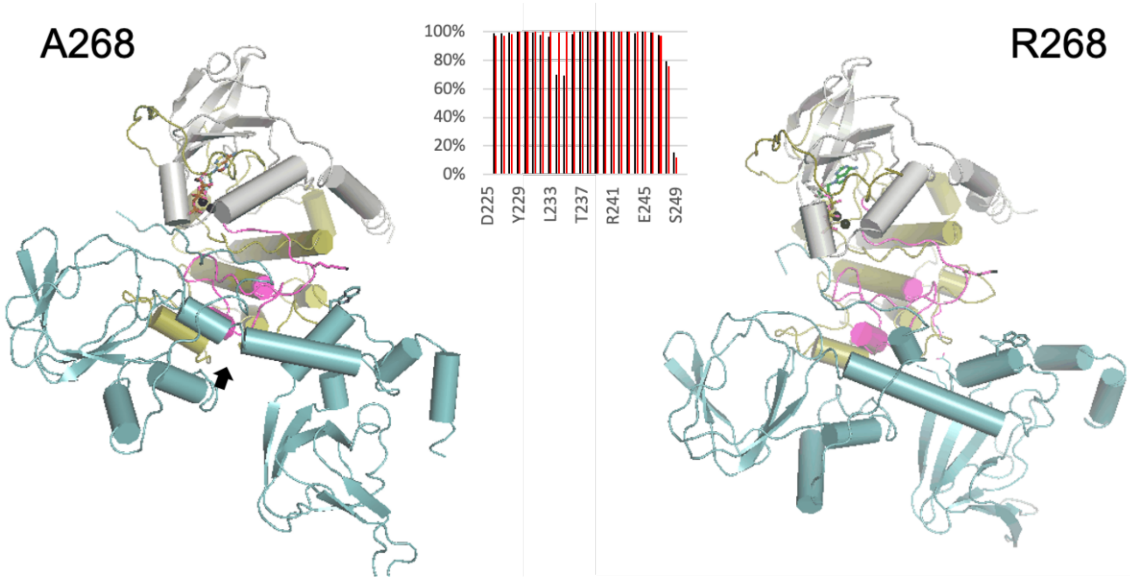

Figure S6.3: Helical propensity and conformational changes in the B/C helix of the R-subunit. Left and Right: Movies from MD simulations of the A268 (left) and R268 (variant, right), showing representative conformations. The N-lobe of the C-subunit is colored gray, the C-lobe is yellow, and the R-subunit is teal. A disruption in the B/C helix is indicated by a black arrow in the A268 variant. **Center:** Bar graph showing the helical propensity of individual residues in the B/C helix, based on DSSP analysis using cpptraj. The y-axis represents the percentage of simulation frames in which each residue adopts an α-helical conformation. Black bars represent the A268; red bars represent the R268 variant. The A268 shows a decrease in helicity near residue 235. Structural model rendered in PyMOL v2.5.

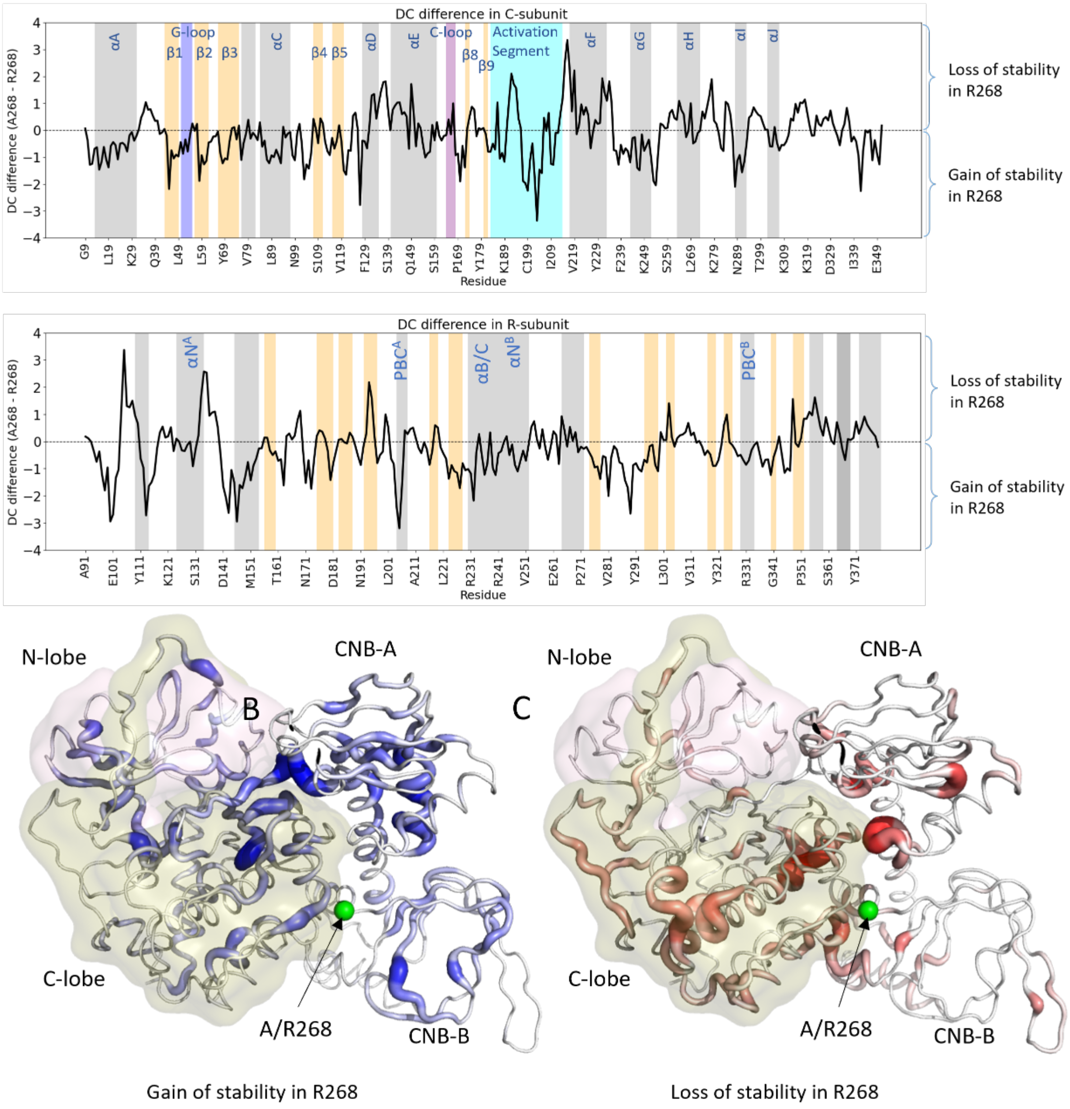

Figure S7: Changes in degree centrality between A268 and R268 variant. The difference in degree centrality (DC) between A268 and R268 (A268 – R268) is plotted for the catalytic (C) and regulatory (R) subunits. Positive values indicate a decrease in stability (i.e., increased dynamics) in the R268 variant, while negative values reflect regions that are more stable (i.e., less dynamic) in R268 compared to A268. Secondary structure elements are indicated with α-helices in gray and β-sheets in light orange. Key functional regions, including the Gly-loop, catalytic loop, and activation segment, are annotated. **Bottom panels:** DC differences are mapped onto the crystal structure (PDB ID: 4DIN). Regions with negative DC differences (A268 > R268), representing areas that are more stable in the R268 variant, are shown in **blue**. Regions with positive DC differences (A268 < R268), indicating increased flexibility or destabilization in the R268 variant, are shown in **red**. Structural model rendered in PyMOL v2.5.

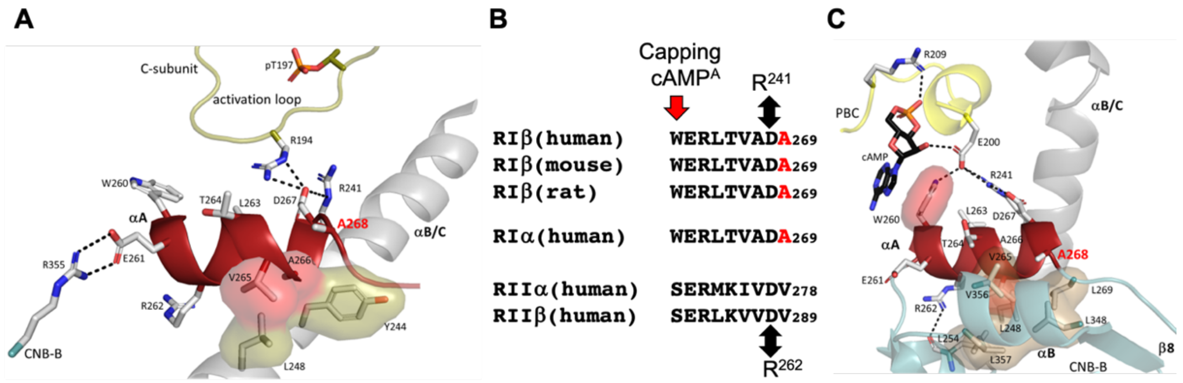

Figure S8: Sequence alignment of mammalian RIβ reveals A268 as the canonical variant. (A) The RIβ holoenzyme (H-form) with Cα shows that the second N3A motif (red) interacts with the activation loop of the C-subunit as well as with the CNB-B domain. The activation loop of Cα (R194) interacts with D267, which in turn positions R241 of the second N3A motif. Additionally, the N3A-E261 interaction with the CNB-B domain (R355) is observed. **(B)** Alignment of RIβ species shows a conserved alanine at position 268, with the capping residues W260 and D267, important for R241 positioning. **(C)** In the cAMP-bound B-form, W260 and D267 in the second N3A motif play a critical role in cAMP binding by interacting with R241 and positioning E200 for high-affinity cAMP binding. If these amino acids are not positioned correctly, the CNB domains become uncoupled, and affinity is reduced in CNB-A. Structural model rendered in PyMOL v2.5. Sequences were retrieved from the UniProt database, and the alignment was generated using the UniProt Align tool (Clustal Omega). UniProt accession numbers used for the alignment are: Human PRKAR1A (P10644), Human PRKAR1B (P31321), Human PRKAR2A (P13861), Human PRKAR2B (P31323), Mouse PRKAR1B (P12849), and Rat PRKAR1B (P81377).
