# Supplementary figures and images for: Functional divergence of protein kinase A regulatory subunit Iβ variants: the importance of N3A motifs in PKA regulation
Source: FEBS J. 2025 Dec 13;293(8):2417–34. doi: 10.1111/febs.70358 (PMC13080237; doi:10.1111/febs.70358)

## Slide 1
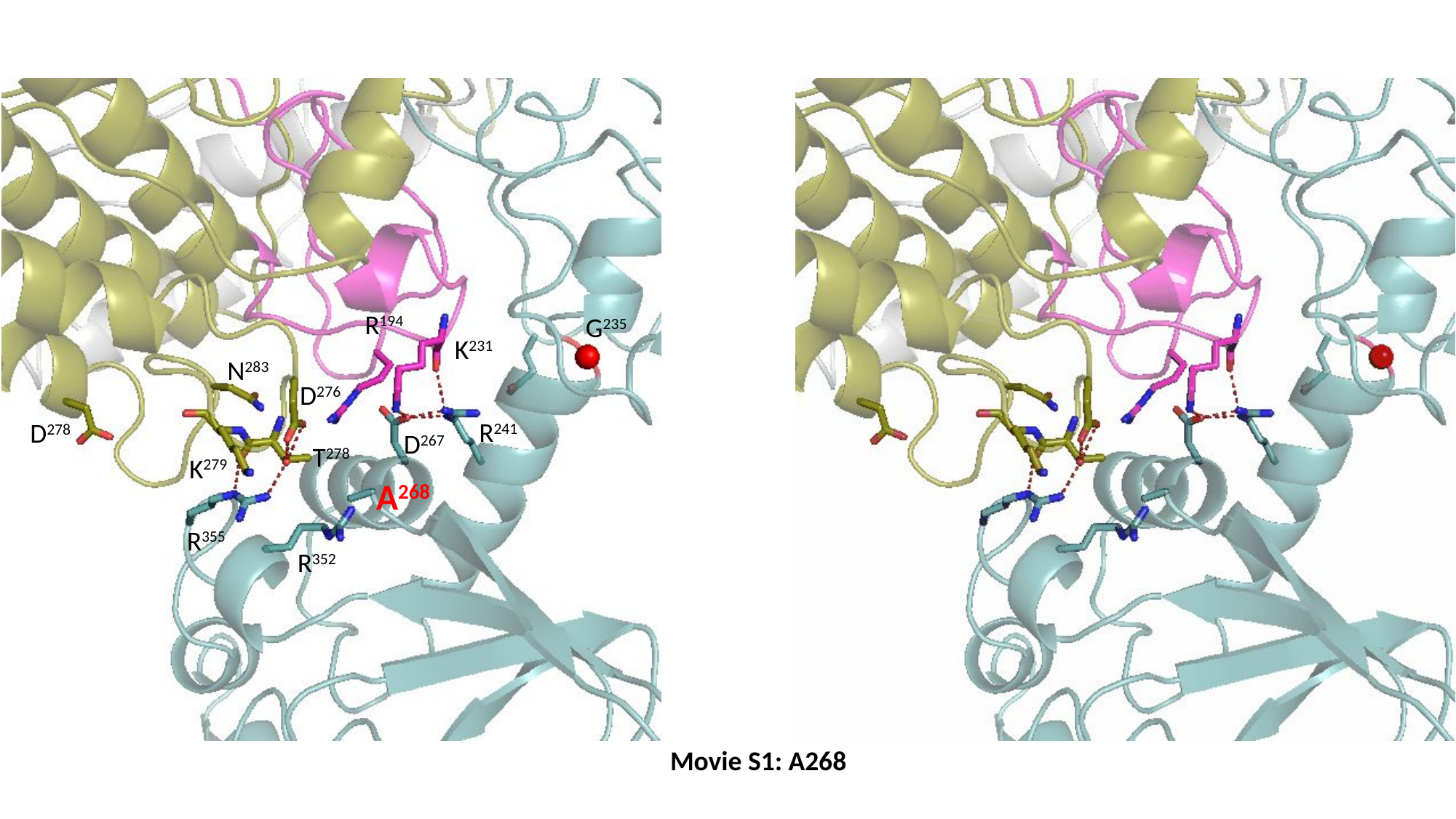

R194
G235
K231
N283
D276
R241
D278
D267
T278
K279
A268
R355
R352
Movie S1: A268

## Slide 2
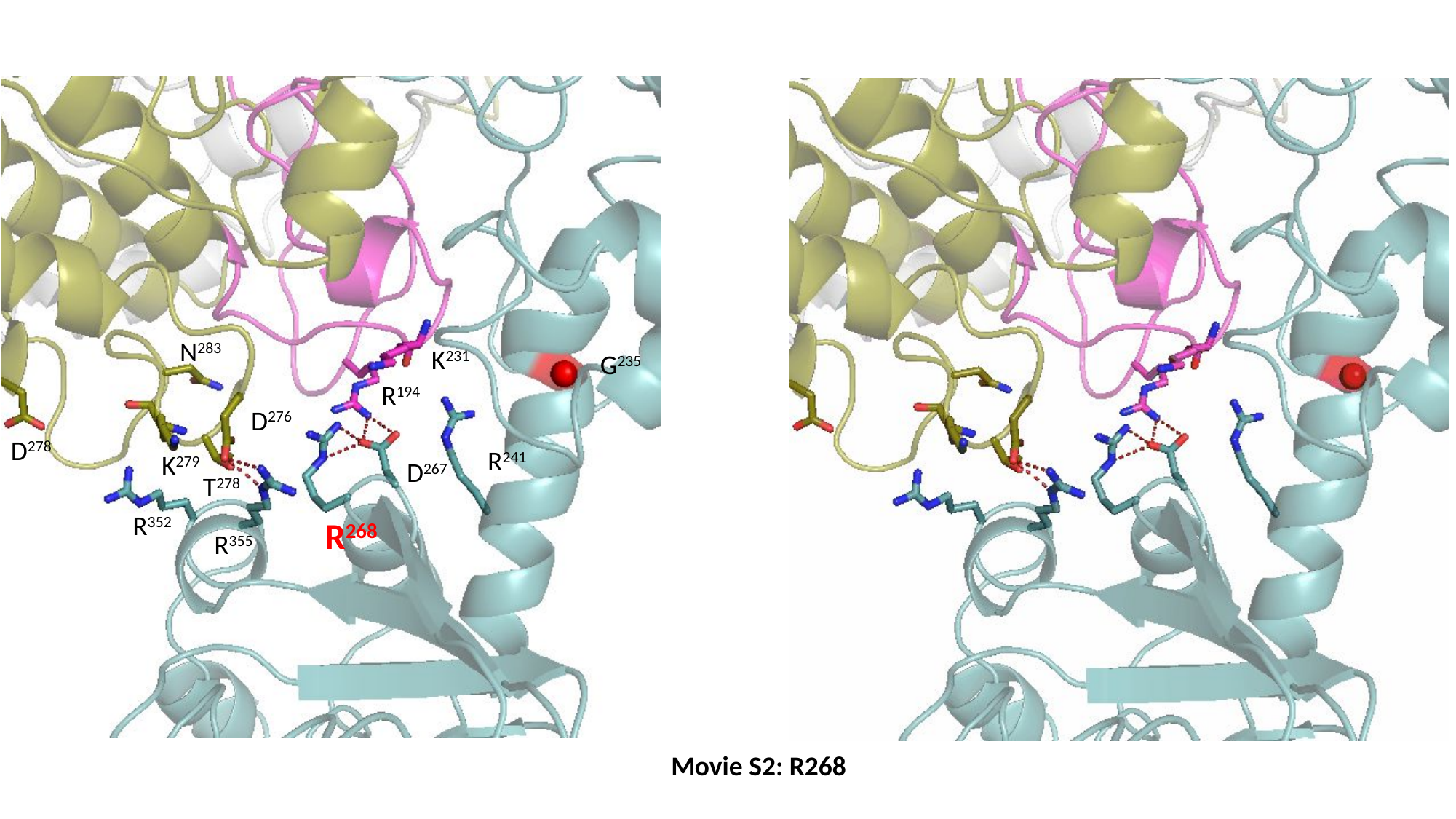

N283
K231
G235
R194
D276
D278
R241
K279
D267
T278
R352
R268
R355
Movie S2: R268

Supplement: Supplementary file 2 — Movie S1. Snapshots from MD simulations of the A268 variant, showing representative conformations and differences in interactions between the R and C subunits. Hydrogen bonds are shown as dashed red lines. Movie S2. Snapshots from MD simulations of the R268 variant, showing representative conformations and differences in interactions between the R and C subunits. Hydrogen bonds are shown as dashed red lines. [file FEBS-293-2417-s001.pptx]
